# Rescue of cochlear vascular pathology prevents sensory hair cell loss in Norrie disease

**DOI:** 10.1073/pnas.2322124121

**Published:** 2024-11-25

**Authors:** Aara Patel, Valda Pauzuolyte, Neil J. Ingham, Yeh Chwan Leong, Wolfgang Berger, Karen P. Steel, Jane C. Sowden

**Affiliations:** ^a^Developmental Biology and Cancer Department, University College London Great Ormond Street Institute of Child Health, University College London, and National Institute for Health and Care Research Great Ormond Street Hospital Biomedical Research Centre, London WC1N 1EH, United Kingdom; ^b^Wolfson Sensory, Pain and Regeneration Centre, King’s College, London SE1 1UL, United Kingdom; ^c^Institute of Medical Molecular Genetics and Zurich Center for Integrative Human Physiology, and Neuroscience Center Zurich, University and ETH Zurich, University of Zürich, Zurich 8057, Switzerland

**Keywords:** cochlea, vascular endothelial cells, NDP, norrin

## Abstract

Variants in the gene *NDP* cause Norrie disease, a severe dual-sensory disorder characterized by congenital blindness due to disrupted retinal vascular development and progressive hearing loss accompanied by sensory hair cell death. *NDP* encodes the secreted signaling molecule norrin. The role of norrin in the cochlea is incompletely understood. We investigated whether the Norrie disease cochlear pathology can be ameliorated in an *Ndp*-knockout (*Ndp*-KO) mouse model by conditional activation of stabilized β-catenin in vascular endothelial cells. We hypothesized that in the cochlea microvasculature, β-catenin is the primary downstream intracellular effector of norrin binding to endothelial cell surface receptors and that restoration of this signaling pathway is sufficient to prevent sensory hair cell death and hearing loss. We show that tamoxifen induction of *Cdh5CreERT2;Ctnnb1^flex3/+^;Ndp-*KO mice stabilizing β-catenin in vascular endothelial cells alone rescued defects in cochlear vascular barrier function, restored dysregulated expression of endothelial cell disease biomarkers (*Cldn5, Abcb1a, Slc7a1,* and *Slc7a5*), and prevented progressive outer hair cell death and hearing loss. Single-cell transcriptome profiling of human cochleas showed *NDP* expression by fibrocytes and glial cells while receptor gene expression (*FZD4, TSPAN12, LRP5,* and *LRP6*) coincided in vascular endothelial cells. Our findings support the conclusion that vascular endothelial cells are a primary target of norrin signaling in the cochlea of mice and humans and restoration of β-catenin regulation of target gene expression within cochlear endothelial cells is sufficient to maintain a cochlear microenvironment critical for hair cell survival.

## Significance

Norrie disease is a form of deaf blindness caused by mutations in *NDP* (Norrin Cystine Knot Growth Factor NDP gene). While vision loss is present at birth, hearing loss has a later onset making it a focus for treatment. Microvasculature and sensory hair cells are sites of pathology in the cochlea. We recently showed that these sites of pathology and progressive hearing loss could be prevented by gene therapy. Here, we investigate the disease mechanism and demonstrate that treating cochlear microvasculature by targeting an intracellular signaling pathway is sufficient to prevent outer hair cell death. This finding increases the potential targets for gene therapy. It highlights the importance of cochlear microvasculature with potential implications for more common forms of hearing loss.

**Fig. 1.**
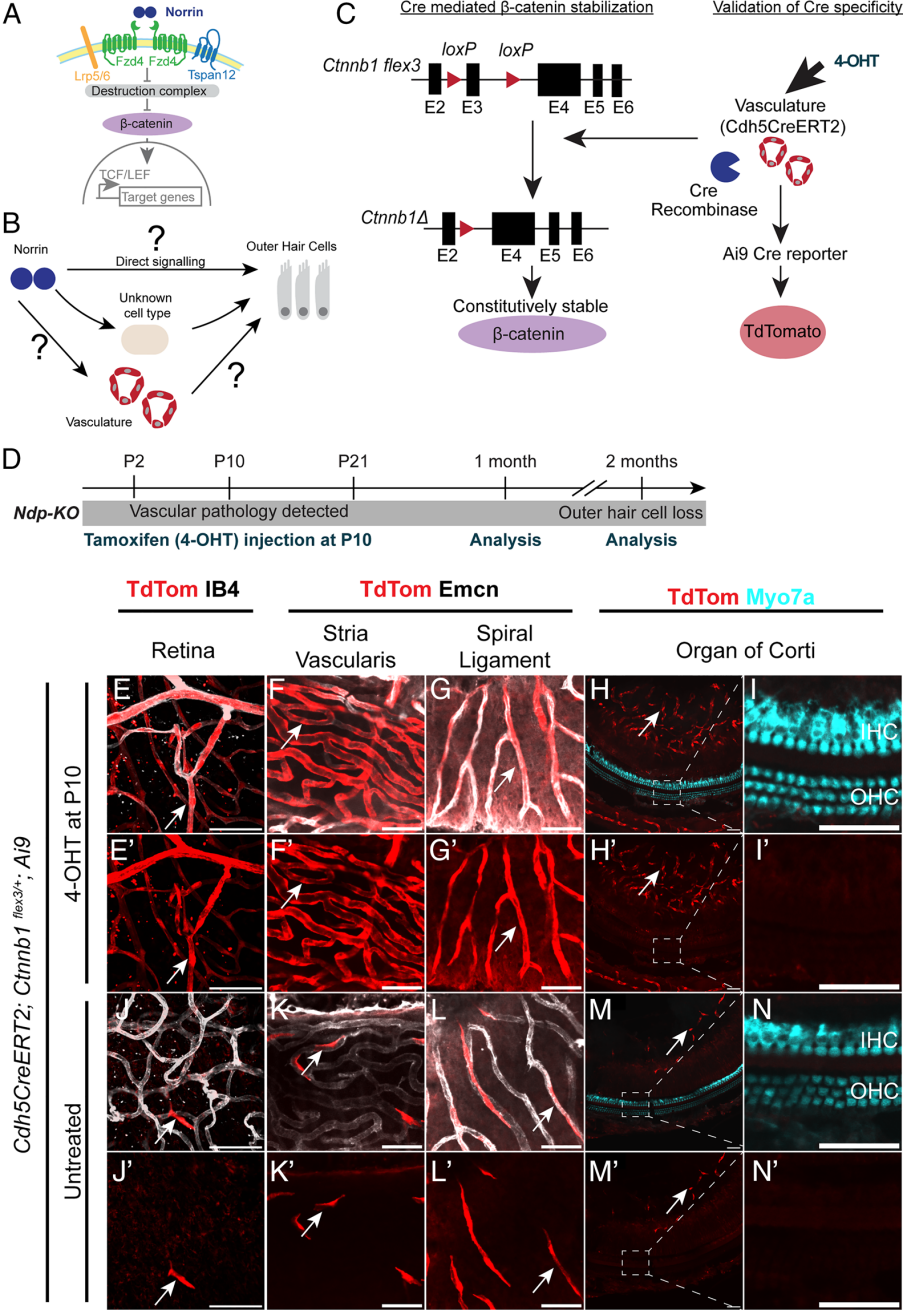
Study design: The principle of β-catenin stabilization in vascular endothelial cells and specificity of Cdh5-Cre expression in the lateral wall and modiolus of the mouse cochlea. (*A*) Schematic showing the norrin signaling pathway. Norrin binds to a receptor complex stabilizing β-catenin which translocates to the nucleus and activates target genes. (*B*) Schematic showing potential mechanisms of Norrin action in the cochlea. (*C*) A tamoxifen-inducible Cre-lox system was used to activate β-Catenin signaling in vascular endothelial cells only. (*D*) Mice were treated with 4-OHT at P10, when the vascular pathology is first detected and before the onset of OHC death and analyses at 1 and 2 mo of age. (*E*–*N*) Cre specificity was tested using the Ai9 Cre reporter. In the presence of 4-OHT, Cre activity was observed in endothelial cells of the retinal vasculature (*E* and *E’*), the stria vascularis (*F* and *F’*), spiral ligament (*G* and *G’*), and modiolus (*H* and *H’*). It was not expressed in the cochlear hair cells (*H*–*I’*). Cre activity was rarely observed in the absence of 4-OHT, only in vascular endothelial cells (*J*–*N’*). 4-OHT treated n = 4, untreated n = 3. [Scale bar: 50 µm (*E*–*N’*).]

The *NDP* gene encodes norrin, a secreted Wnt analogue. Norrin acts through a receptor complex consisting of FZD4, LRP5 or LRP6, and TSPAN12 (1–4). Binding of norrin dimer to FZD4 and LRP5/6 causes the formation of a ternary complex, enhanced by TSPAN12 (3, 5), inhibiting the degradation complex that normally turns over β-catenin, thus stabilizing cytoplasmic β-catenin that in turn translocates to the nucleus and modulates the expression of downstream genes (1, 6). (Fig. 1*A*). An in vitro study also showed interaction of norrin with LGR4 activating the intracellular canonical WNT pathway (7); however, the in vivo significance of this is unknown.

Variants in *NDP* cause Norrie disease, an X-linked recessive dual-sensory disorder (8, 9), or a milder ocular condition, familial exudative vitreoretinopathy (FEVR) (10). Boys with Norrie disease are born blind due to malformation of the retinal vasculature, absence of deep retinal vasculature, retinal exudation, superficial neovascularization, and tractional retinal detachment (11). The majority also develop progressive hearing loss, and a proportion present with seizures and cognitive impairment (9, 12). Mice lacking any functional copy of the homologous gene *Ndp* [*Ndp^tm1Wbrg^*/^Y^(*Ndp*-KO)] closely recapitulate human Norrie disease. They lack deep retinal vasculature, develop superficial retinal neovascularization, have an impaired blood–retina barrier (13–15), and develop progressive hearing loss (16). Mutations in FZD4, TSPAN12, or LRP5/6 cause FEVR (17–20), phenocopying NDP mutations. These are characterized by peripheral retinal avascularization and retinal vascular exudation, albeit without reported hearing loss. *Fzd4*-null mice have a severe Norrie disease-like retinal vasculature and affected hearing, with abnormal cochlear vasculature and hair cell loss (1). *Tspan12*-null mice have abnormal retinal and cochlear vasculature (3, 21).

In the retina, vascular endothelial cells are the primary site of Norrie disease pathology (22). Deletion of *Ctnnb1* (encoding β-catenin) in just endothelial cells caused a retinal phenotype similar to that of the *Ndp*-KO mouse. Conversely, the phenotype was prevented by endothelial-specific stabilization of β-catenin (6). Norrin also contributes to maintenance of the cerebellar vasculature along with Wnt7a/Wnt7b signaling and converging on Fzd4 and β-catenin stabilization (23). The retinal and brain vasculatures share barrier properties regulating transport of molecules across vessel walls (24).

Cochlear Norrie disease pathology is more complex. A postmortem study of a Norrie disease patient reported degeneration of the stria vascularis, hair cells, and cochlear neurons and proliferation of connective tissue (25). An initial study in *Ndp*-KO mice (*Ndp^tm1Wbrg^*) analyzed at 3, 6, and 15 mo identified the stria vascularis as the primary lesion site and reported degeneration of sensory hair cells and the spiral ganglion (16). A subsequent study of Norrie disease mice (*Ndp^tm2Nat^*) reported that abnormal outer hair cell (OHC) gene expression as early as postnatal day 3 (P3) and death of OHCs starting at 2 mo occurred prior to and independently of vascular defects that were only apparent at 6 mo. Furthermore, increasing the level of β-catenin in hair cells rescued hearing leading to the conclusion that direct norrin signaling is essential for maturation and survival of OHCs (26). In contrast, we recently showed that the stria vascularis and spiral ligament capillaries had an abnormal morphology as early as P10 (*Ndp^tm1Wbrg^*) (13, 27) and abnormal barrier function at P20. OHC death was apparent by 1 mo of age, worsening by 2 mo of age with functional hearing loss (27). By 3 mo, responses to low-frequency auditory stimuli were significantly impaired, as measured by distortion product otoacoustic emissions (DPOAEs) and auditory brainstem responses (ABR), corresponding to hair cell loss in the mid-apical region of the organ of Corti (28). Transcriptomic analysis of the mouse cochlea identified genes whose expression was dysregulated in the absence of norrin signaling. These included the vascular endothelial tight junction gene *Cldn5* and endothelial transporters *Abcb1a, Slc7a1, Slc7a5,* and *Plvap*, emphasizing the involvement of the cochlear vascular barrier in Norrie disease. Restoration of secreted norrin in the cochlea by means of an AAV-mediated gene therapy administered as late as P30 prevented further OHC loss, suggesting that early norrin signaling was not essential for OHC maturation and function (28)

Expression of *Ndp* in the mouse cochlea has been described both in the lateral wall and in the zone between the spiral ganglion and organ of Corti (26, 29), suggesting multiple potential targets of norrin signaling. Following our observations of an early vascular pathology in the *Ndp-KO* model (*Ndp^tm1Wbrg^*), in this study, we aimed to resolve the primary target of norrin signaling in the cochlea that is essential for normal cochlea tissue homeostasis. We hypothesized that if OHC death is secondary to vascular defects, rather than OHC or other cochlear cells requiring direct norrin signaling, then rescuing the vascular defects would indirectly prevent OHC death (Fig. 1*B*). We tested the hypothesis in *Ndp*-KO mice by genetically stabilizing β-catenin, the intracellular mediator of norrin signaling, in vascular endothelial cells alone, expecting that this would recapitulate the effect of norrin on cochlea endothelial cells. We assessed whether this intervention rescued the vascular phenotype and was sufficient to prevent hair cell death in the absence of secreted norrin. Finally, to determine whether the mechanism of Norrie cochlear pathology demonstrated in the mouse cochlea was compatible with the human cochlea, we used single-cell transcriptomic analysis to profile the expression of *NDP* and norrin receptor and target genes in the human cochlea.

## Results

### Conditional Stabilization of β-Catenin in Vascular Endothelial Cells.

The Cre/loxP system is widely used to achieve cell type–specific gene induction or suppression (30). The mouse *Ctnnb1^flex3^* allele carries loxP sequences flanking the in-frame exon 3, which codes for sites of GSK-3β phosphorylation that promote β-catenin ubiquitination and degradation (31). The *Cdh5CreERT2* inducible Cre allele (32, 33) encodes a Cre-ERT2 fused sequence under the control of the promoter sequence of *Cdh5* and expresses cytoplasmic CreERT2 fusion protein in vascular endothelial cells. A mouse line homozygous for both the *Cdh5CreERT2* and *Ctnnb1^flex3^* alleles was generated. Immunostaining in these mice, using an anti-estrogen receptor antibody to detect Cre-ERT2, verified specificity of the *Cdh5* promoter for endothelial cells in the cochlear vasculature (*SI Appendix*, Fig. S1, yellow arrowheads). On tamoxifen induction, CreERT2 translocates to the nucleus and causes recombination at loxP sequences, deleting exon 3 of the *Ctnnb1* flex3 allele and generating a stabilized form of β-catenin in vascular endothelial cells, resistant to phosphorylation by GSK-3β and subsequent degradation (Fig. 1*C*). Cre was induced in *Cdh5-CreERT2/Cdh5-CreERT2; Ctnnb1^flex3/flex3^* mice by intraperitoneal administration of 4-OHT (4-Hydroxy tamoxifen) at postnatal day (P)10. Immunostaining using anti-β-catenin antibody showed β-catenin accumulation confined to vascular endothelial cells (*SI Appendix*, Figs. S2 and S3).

To further verify the endothelial cell specificity of Cre recombination activity in the cochlea, we crossed the Cdh5CreERT2 homozygous; *Ctnnb1^flex3/flex3^* mouse line with Ai9 Cre reporter homozygous mice. The Ai9 allele carries a CAG promoter-driven TdTomato fluorescent protein gene preceded by a lox-P-flanked STOP cassette construct (34). In cells expressing Cre recombinase, permanent deletion of the STOP cassette results in TdTomato expression (Fig. 1*C*). Cre was induced by intraperitoneal administration of 4-OHT to *Cdh5CreERT2; Ctnnb1^flex3/+^; Ai9* pups at P10 when vascular abnormalities were first observed in the *Ndp*-KO mouse cochlea (27). Analyses were performed at 1 mo, when vascular pathology was apparent, but before cochlear OHC death, and at 2 mo when OHC death and hearing loss have progressed (Fig. 1*D*).

At 1 mo, TdTomato was detected in vascular endothelial cells of retinal vessels and the stria vascularis, spiral ligament, and the modiolus of the cochlea vessels (Fig. 1 *E*–*I’* and *SI Appendix*, Figs. S4 and S5). Importantly, TdTomato was not detected in outer or inner hair cells (IHCs), supporting cells, nor any other cell types in the organ of Corti or lateral wall (Fig. 1 *H*–*I*’ and *SI Appendix*, Fig. S5 *C* and *C’*). In untreated littermate mice, also carrying all three alleles, a small number of vascular endothelial cells expressed TdTomato (Fig. 1 *J*–*N’* and *SI Appendix*, Figs. S4 and S5). However, again, there was no expression in the hair cells (Fig. 1 *M*–*N’* and *SI Appendix*, Fig. S5 *D* and *D’*). In the uninduced state, Cre recombination activity (i.e., CreERT2 activity without 4-OHT binding) was observed rarely and only in endothelial cells (Fig. 1 and *SI Appendix*, Figs. S4 and S5). Together, these data indicate that the *Cdh5CreERT2* model is a suitable tool to activate β-catenin signaling specifically in vascular endothelial cells while leaving cochlear hair cells and other cochlea cells unaffected.

For analyses on the effect on Norrie disease pathology, male *Cdh5CreERT2 homozygous;Ctnnb1^flex3/flex3^* mice were crossed with *Ndp^+/−^* heterozygous female mice. *Cdh5CreERT2; Ctnnb^flex3/+^; Ndp-KO* or *Cdh5CreERT2;Ctnnb^flex3/+^*;*WT* pups were treated with 4-OHT at P10, to induce vascular β-catenin stabilization; untreated *Cdh5CreERT2;Ctnnb1^flex3/+^; Ndp-KO* or *Cdh5CreERT2;Ctnnb1^flex3/+^; WT* mice were used as control groups (Fig. 1*D*).

### Early Stabilization of β-Catenin in Vascular Endothelial Cells Rescued *Ndp-*KO Retinal Vascular Morphology and Barrier Function.

**Fig. 2.**
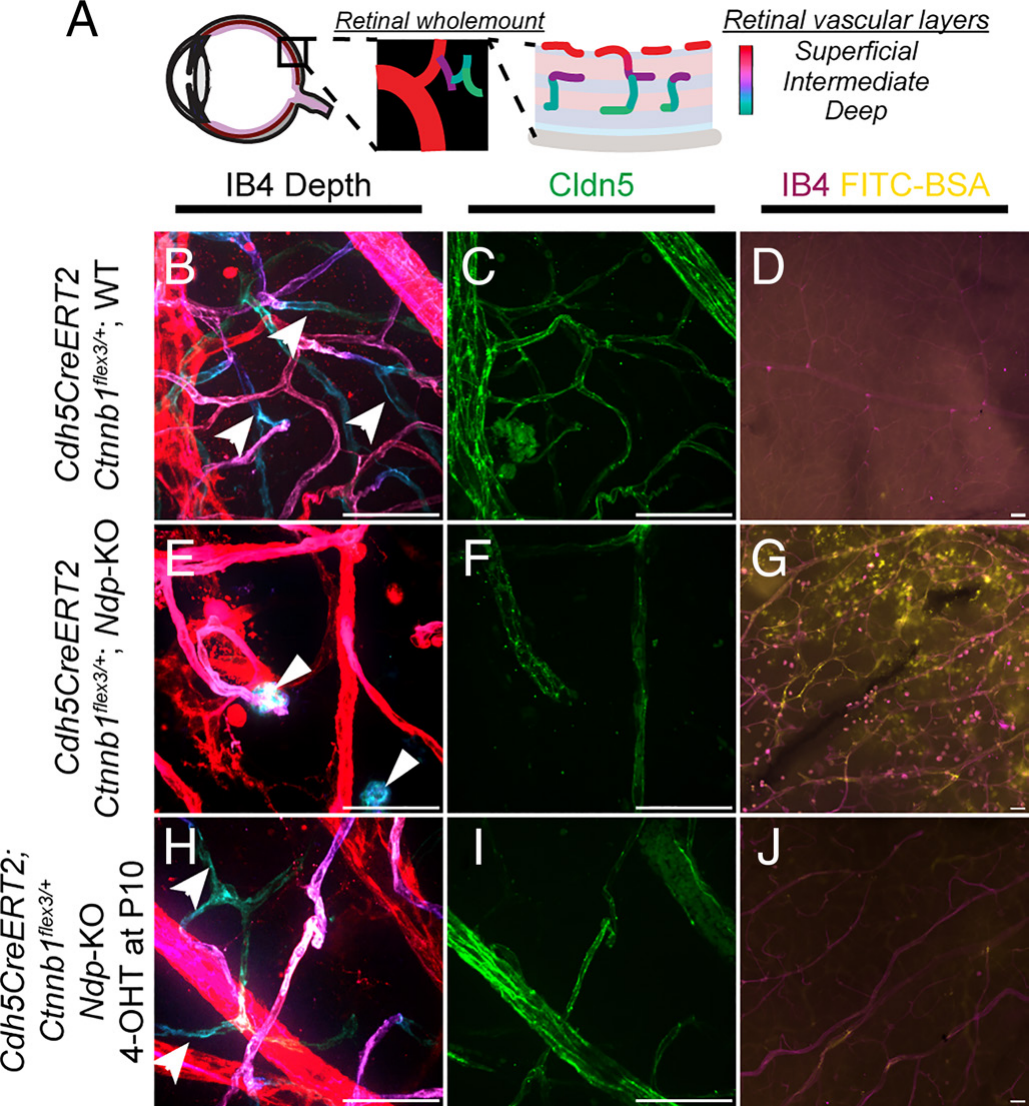
Analysis of rescue of retinal vasculature at 2 mo in *Ndp*-KO mice by vascular β-catenin stabilization at P10. (*A*) Schematic showing the retinal vasculature consisting of three layers. (*B*–*D*) WT mice had three vascular layers (arrows indicate deep vessels), with high Cldn5 expression (*C*) which prevented the leakage of intravenous FTIC-BSA (*D*). (*E*–*G*) *Ndp*-KO mice had a superficial vascular layer (*E*) with protrusions into the retina (arrowheads), reduced Cldn5 expression (*F*), and a defective vascular barrier leading to leakage of intravenous FITC-BSA (*G*). (*H*–*J*) *Ndp*-KO mice with vascular endothelial β-catenin stabilization formed deep retinal vessels (arrows), restored Cldn5 expression (*I*), and prevented FITC-BSA (*J*). Vascular morphology *Cdh5CreERT2;Ctnnb1^flex3/+^;*WT n = 6, *Cdh5CreERT2;Ctnnb1^flex3/+^;Ndp*-KO = 5, 4-OHT-treated *Cdh5CreERT2;Ctnnb1^flex3/+^;Ndp*-KO n = 11; Cldn5 expression *Cdh5CreERT2;Ctnnb1^flex3/+^;*WT n = 5,*Cdh5CreERT2;Ctnnb1^flex3/+^;Ndp*-KO = 3, 4-OHT-treated *Cdh5CreERT2;Ctnnb1^flex3/+^; Ndp*-KO n = 10; vascular barrier function *Cdh5CreERT2;Ctnnb1^flex3/+^;*WT n = 2, *Cdh5CreERT2;Ctnnb1^flex3/+^;Ndp*-KO n = 2, 4-OHT-treated *Cdh5CreERT2;Ctnnb1^flex3/+^;Ndp*-KO n = 4. [Scale bar: 50 µm (*B*–*J*).]

At 2 mo of age, *Cdh5CreERT2;Ctnnb^flex3/+^;*WT mice had three complete retinal capillary layers (Fig. 2*A*)—superficial, intermediate, and deep, with strong expression of the tight junction protein Cldn5 (Fig. 2 *B* and *C*). FITC-BSA, a fluorescent tracer, was injected intravenously 3 h prior to killing the mice. In WT mice, it did not leak out of retinal capillaries indicating an intact blood–retina barrier (Fig. 2*D*). Untreated *Cdh5CreERT2;Ctnnb1^flex3/+^;Ndp*-KO mice at the same age had only a thick superficial layer of vessels (Fig. 2 *E* and *F*) located above the ganglion cell layer, with reduced Cldn5 expression. FITC-BSA showed extensive leakage and deposition in the retina (Fig. 2*G*). Vascular β-catenin stabilization in 4-OHT-treated *Cdh5CreERT2;Ctnnb1^flex3/+^; Ndp*-KO mice restored the formation of deep capillary layers and expression of Cldn5 (Fig. 2 *H* and *I*). Leakage of intravenous FITC-BSA was prevented (Fig. 2*J*). These findings are consistent with a previous study, which used another endothelial Cre driver (*Pdgfb*-*CreER*) to stabilize β-catenin at P3 and demonstrated a correction of retinal vascular morphology and blood–retina barrier function (6) indicating that vascular β-catenin stabilization in our system has an expected effect.

### Stabilization of β-Catenin in Cochlear Vascular Endothelial Cells Rescued the Expression of Vascular Disease Markers.

We recently showed that *Cldn5* was down-regulated in the *Ndp*-KO cochlea from as early as P20, while *Plvap* was up-regulated from 1 mo onward (27). RNAseq analysis identified additional disease biomarker genes in the *Ndp*-KO at 2 mo: *Abcb1a, Slc7a1, Slc7a5, and Sox17* were down-regulated; *Clu, Ceacam16, and Nr1h4* were up-regulated (28). Timeline analysis of these disease marker genes by qRT-PCR in *WT* and *Ndp*-KO mouse cochleas at P10, P20, 1 mo, and 2 mo identified early changes in expression of endothelial markers (*SI Appendix*, Fig. S6). Genes associated with endothelial cell function (35–37) *Abcb1, Slc7a1, and Slc7a5* were significantly down-regulated in *Ndp*-KO mice compared to WT from P20 onward, similar to *Cldn5* (*SI Appendix*, Fig. S6 *A*–*C*), whereas *Nr1h4*, *Clu,* and *Ceacam16* [expressed by multiple cell types (38–40)] were up-regulated only at later timepoints (*SI Appendix*, Fig. S6 *E*–*G*). *Sox17* was transiently down-regulated, at P20 but not at later timepoints (*SI Appendix*, Fig. S6 *D*); Sox17 encodes a transcription factor gene up-regulated in response to norrin signaling in retinal endothelial cells (2).

**Fig. 3.**
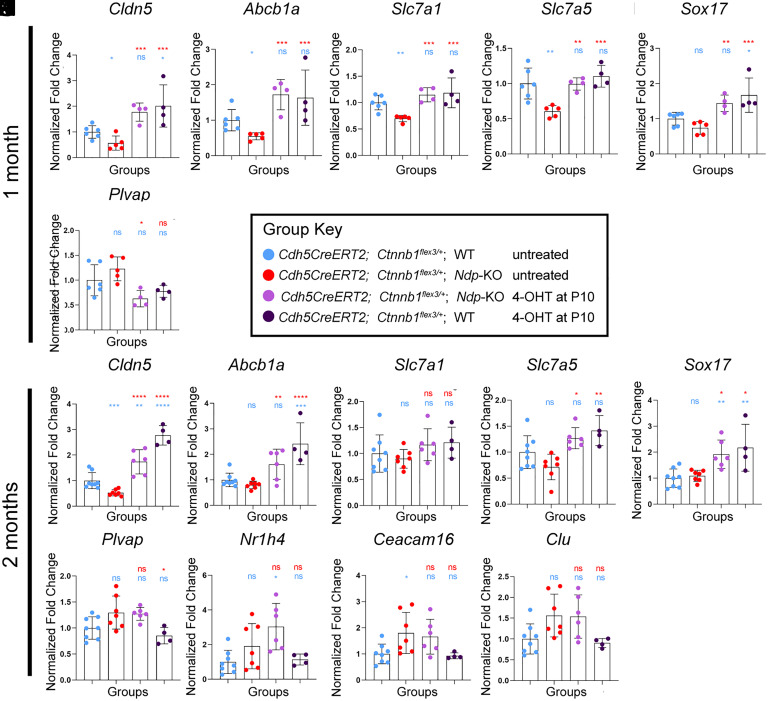
qRT-PCR analysis shows normalization of the expression of vascular and nonvascular genes in the cochlea at 1 and 2 mo after vascular endothelial β-catenin stabilization at P10. (*A*–*F*) Expression of cochlear Norrie disease biomarker genes in treated and control mice at 1 mo by qRT-PCR. (*G*–*O*) Expression of disease biomarker genes in treated and control mice at 2 mo by qRT-PCR analysis. qRT-PCR 1 mo: *Cdh5CreERT2;Ctnnb1^flex3/+^;*WT n = 6, *Cdh5CreERT2;Ctnnb1^flex3/+^; Ndp*-KO n = 5, 4-OHT-treated *Cdh5CreERT2;Ctnnb1^flex3/+^;Ndp*-KO n = 4, 4-OHT-treated *Cdh5CreERT2;Ctnnb1^flex3/+^;*WT n = 4; qRT-PCR 2 mo: *Cdh5CreERT2;Ctnnb1^flex3/+^;*WT n = 8, *Cdh5CreERT2;Ctnnb1^flex3/+^;Ndp*-KO n = 7, 4-OHT-treated *Cdh5CreERT2;Ctnnb1^flex3/+^;Ndp*-KO n = 6, 4-OHT-treated *Cdh5CreERT2;Ctnnb1^flex3/+^;*WT n = 4. Data represent mean ± SD. Data represent mean ± SD. Analyzed with one-way ANOVA with Sidak’s post hoc test, *P ≤ 0.05, **P ≤ 0.01, ***P ≤ 0.001, and ****P ≤ 0.0001.

Whole cochlea lysates of 1- and 2-mo-old 4-OHT-treated and control mice were analyzed by qRT-PCR (Fig. 3). At 1 mo, *Cldn5*, *Abcb1a*, *Slc7a1,* and *Slc7a5* were significantly down-regulated in untreated *Cdh5CreERT2;Ctnnb1^flex3/+^;Ndp-KO* mice (Fig. 3 *A*–*D*, red) compared to *Cdh5CreERT2;Ctnnb1^flex3/+^;WT* (Fig. 3 *A*–*D*, blue). *Sox17* showed a similar trend (Fig. 3*E*, red versus blue). Vascular β-catenin stabilization by 4-OHT treatment caused a significant upregulation of all these genes in treated *Cdh5CreERT2; Ctnnb1^flex3^*^/+^;*Ndp*-KO mice light purple) (Fig. 3 *A*–*E*) compared to untreated *Cdh5CreERT2;Ctnnb1^flex3^*^/+^;*Ndp*-KO. *Plvap* was significantly down-regulated by vascular β-catenin stabilization (Fig. 3*F*, light purple).

At 2 mo, *Cldn5* was still significantly down-regulated in untreated *Cdh5CreERT2;Ctnnb1^flex3^*^/+^;*Ndp*-KO mice compared to untreated *Cdh5CreERT2;Ctnnb1^flex3^*^/+^;*WT* (Fig. 3*G*). *Abcb1a, Slc7a1,* and *Slc7a5* showed a similar trend of downregulation in the *Ndp*-KO (Fig. 3 *H*–*J*), while *Sox17* did not (Fig. 3*K*). Vascular β-catenin stabilization caused a significant upregulation of *Cldn5*, *Abcb1a*, *Slc7a5,* and *Sox17* in treated *Cdh5CreERT2;Ctnnb1^flex3^*^/+^;*Ndp*-KO mice consistent with the observations at 1 mo. At 2 mo, *Plvap* and nonvascular late onset disease biomarker genes *Nr1h4*, *Ceacam16*, and *Clu* did not respond to treatment (Fig. 3 *L*–*O*).

Overall, *Cldn5*, *Abcb1a*, *Slc7a1* and *Slc7a5,* and *Sox17* showed the clearest response to treatment, consistent with β-catenin stabilization having an effect on vascular barrier function and transport of biomolecules in the cochlea.

Control *Cdh5CreERT2;Ctnnb1^flex3^*^/+^;*WT* mice with 4-OHT treatment (Fig. 3 *A*–*O*, dark purple) were also analyzed to determine the effects of vascular β-catenin stabilization in a WT background (*Ndp*^+/Y^). Notably, *Cldn5*, and *Sox17* levels at 1 and 2 mo (Fig. 3 *A*, *E*, *G*, and *K*, dark purple) and *Abcb1a* at 2 mo (Fig. 3*H*) were significantly elevated after β-catenin stabilization consistent with a dose–response effect.

### Stabilization of β-Catenin in Vascular Endothelial Cells Rescued Morphology and Barrier Function of the *Ndp*-KO Cochlear Vasculature.

**Fig. 4.**
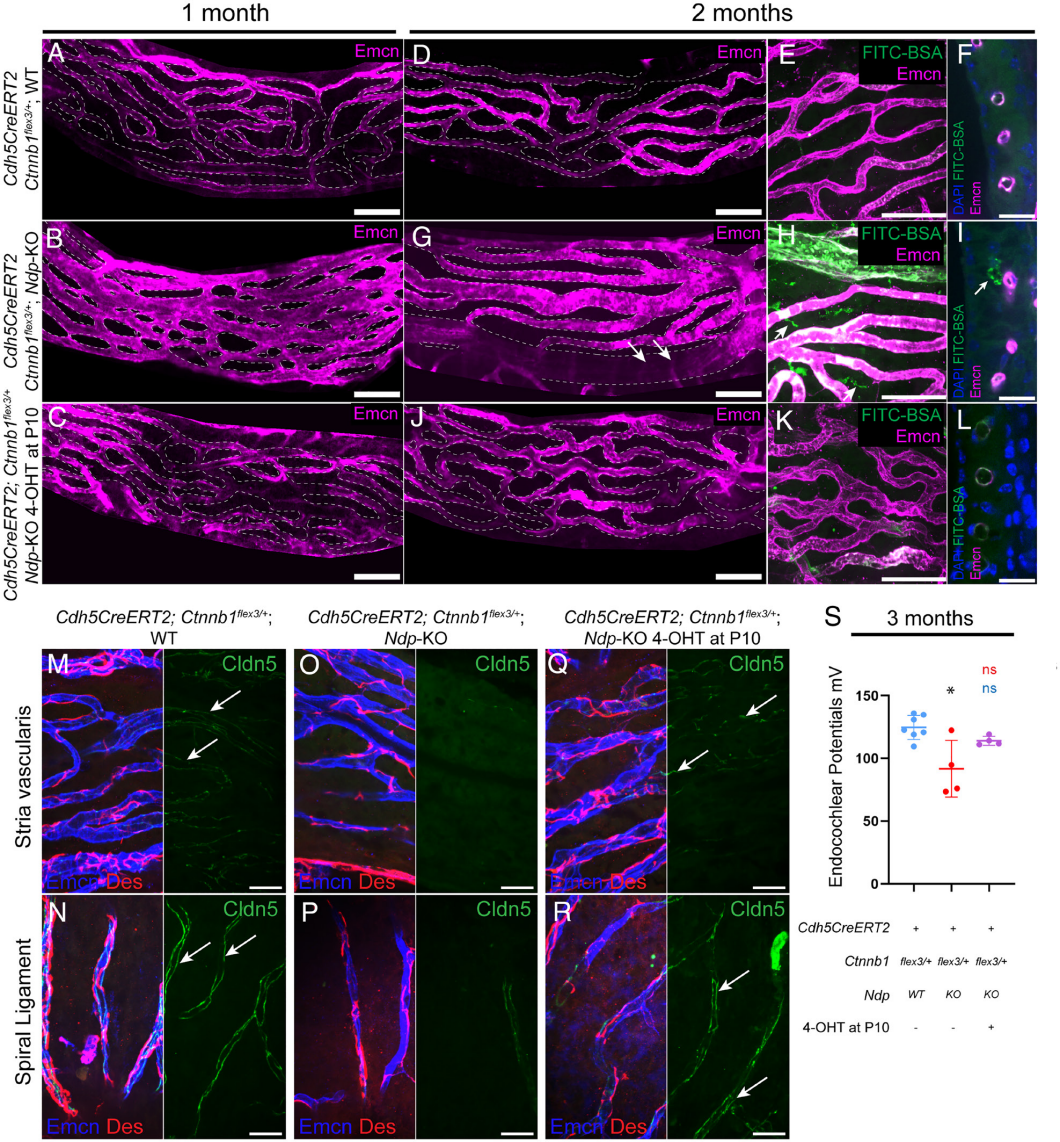
Analysis of lateral walls and endocochlear potential shows that cochlear vasculature of *Ndp*-KO mice was rescued by β-catenin stabilization. (*A*–*C*) Lateral wall whole mounts showing apical stria vascularis capillary morphology by anti-endomucin immunostaining at 1 mo. (*D*–*L*) Wholemounts (*D*, *E*, *G*, *H*, *J*, and *K*) and vibratome sections (*F*, *I*, and *L*) of the lateral wall showing stria vascularis capillary morphology by anti-endomucin immunostaining and barrier function by intravascular FITC-BSA at 2 mo. FITC-BSA localization outside vessels (*H* and *I*) was consistent with a malfunctioning vascular barrier in untreated *Cdh5CreERT2; Ctnnb1^flex3/+^*; *Ndp*-KO mice. (*M*–*R*) Localization of tight junction protein Cldn5 at 2 mo of age. Untreated *Cdh5CreERT2; Ctnnb1^flex3/+^; Ndp*-KO mice had reduced Cldn5 (see also *SI Appendix*, Fig. S7 for single-channel images). (*S*) Endocochlear potential in treated and control mice at 3 mo. Vascular morphology 1 mo *Cdh5CreERT2;Ctnnb1^flex3/+^;*WT n = 3, *Cdh5CreERT2;Ctnnb1^flex3/+^;Ndp*-KO = 6, 4-OHT-treated *Cdh5CreERT2;Ctnnb1^flex3/+^;Ndp*-KO n = 3; vascular morphology 2 mo *Cdh5CreERT2;Ctnnb1^flex3/+^;*WT n = 6, *Cdh5CreERT2;Ctnnb1^flex3/+^;Ndp*-KO = 9, 4-OHT-treated *Cdh5CreERT2;Ctnnb1^flex3/+^;Ndp*-KO n = 9; vascular barrier function 2 mo *Cdh5CreERT2;Ctnnb1^flex3/+^;*WT n = 4, *Cdh5CreERT2;Ctnnb1^flex3/+^;Ndp*-KO n = 6, 4-OHT-treated *Cdh5CreERT2;Ctnnb1^flex3/+^;Ndp*-KO n = 5; Cldn5 localization 2 mo *Cdh5CreERT2;Ctnnb1^flex3/+^;*WT n = 3, *Cdh5CreERT2;Ctnnb1^flex3/+^;Ndp*-KO n = 5, 4-OHT-treated *Cdh5CreERT2;Ctnnb1^flex3/+^;Ndp*-KO n = 4; Endocochlear potential 3 mo *Cdh5CreERT2;Ctnnb1^flex3/+^;*WT n = 7, *Cdh5CreERT2;Ctnnb1^flex3/+^;Ndp*-KO n = 4, 4-OHT-treated *Cdh5CreERT2;Ctnnb1^flex3/+^;Ndp*-KO n = 4. (*S*) analyzed with one-way ANOVA with Sidak’s post hoc test, *P ≤ 0.05, **P ≤ 0.01, ***P ≤ 0.001, and ****P ≤ 0.0001. [Scale bars: 50 µm (*A*–*E*, *G*, *H*, *J*, and *K*) 20 µm (*F*, *I*, *L*, and *N*–*R*).]

The lateral wall vasculature was analyzed by endomucin immunostaining at 1 (Fig. 4 *A*–*C*) and 2 (Fig. 4 *D*–*L*) mo. We previously identified vascular malformations as early as P10 in the *Ndp*-KO which progressively worsened (27).

At 1 mo, strial capillaries in the *Cdh5CreERT2;Ctnnb1^flex3^*^/+^;*WT* lateral wall formed an evenly distributed network (Fig. 4*A*). In the untreated *Cdh5CreERT2;Ctnnb1^flex3^*^/+^; *Ndp*-KO mice, they were enlarged and fused, with the abnormality most evident in the apical region of the cochlea (Fig. 4*B*), consistent with previous observations (27). This change was prevented by vascular β-catenin stabilization in treated *Cdh5CreERT2;Ctnnb1^flex3^*^/+^;*Ndp*-KO and an even network of capillaries was maintained (Fig. 4*C*).

Similarly, at 2 mo, strial capillaries of untreated *Cdh5CreERT2; Ctnnb1^flex3^*^/+^;*WT* mice formed an evenly distributed network (Fig. 4*D*). FITC-BSA, injected intravenously to demonstrate vascular barrier integrity, did not leak into the intercapillary spaces of the stria vascularis (Fig. 4*E*). In sections, it was not visible outside the vessel lumen or walls (Fig. 4*F*). In the untreated *Cdh5CreERT2;Ctnnb1^flex3^*^/+^;*Ndp*-KO lateral wall, strial capillaries showed elongated capillary segments and abnormal diversification into thick vessels with low anti-endomucin staining (Fig. 4*G*, arrows). FITC-BSA was visible in intercapillary spaces (Fig. 4*H*, arrow) and outside the capillary lumen and walls in sections (Fig. 4*I*, arrow) indicating a compromised vascular barrier. In treated *Cdh5CreERT2;Ctnnb1^flex3^*^/+^;*Ndp*-KO mice, the remodeling of stria vascularis capillaries was prevented (Fig. 4*J*) with the capillary network resembling the untreated WT rather than *Ndp*-KO. FITC-BSA did not leak into intercapillary spaces (Fig. 4 *K* and *L*).

*Ndp*-KO mice have reduced Cldn5 protein localization at endothelial tight junctions and abnormal pericyte coverage of stria vascularis and spiral ligament capillaries (27). Consistent with this, stria vascularis and spiral ligament capillaries of 2-mo-old untreated *Cdh5CreERT2;Ctnnb1^flex3^*^/+^;*WT* mice showed anti-Cldn5 labeling (green) at endothelial cell junctions (Fig. 4 *M* and *N*, arrows and *SI Appendix*, Fig. S7 *A’’* and *D’’*). Anti-Desmin immunostaining showed an even pericyte coverage (*SI Appendix*, Fig. S7 *A’’’* and *D’’’*, red). In untreated *Cdh5CreERT2;Ctnnb1^flex3^*^/+^;*Ndp*-KO capillaries, anti-Cldn5 labeling was nearly absent (Fig. 4 *O* and *P* and *SI Appendix*, Fig. S7 *B’’* and *E’’*) and coverage by pericytes (*SI Appendix*, Fig. S7 *B’’’* and *E’’*, red) was reduced. This phenotype was more pronounced in spiral ligament capillaries. In 4-OHT-treated *Cdh5CreERT2; Ctnnb1^flex3^*^/+^;*Ndp*-KO mice, anti-Cldn5 labeling (green) (Fig. 4 *Q* and *R* arrows and *SI Appendix*, Fig. S7 *C’’* and *F’’*) and pericyte coverage were restored (*SI Appendix*, Fig. S7 *C’’’* and *F’’’*). Also, control *Cdh5CreERT2;Ctnnb1^flex3/+^;* WT mice following 4-OHT treatment did not show any abnormality in vascular morphology of the stria vascularis and spiral ligament and Cldn5 appeared correctly localized (*SI Appendix*, Fig. S8).

An intact vascular barrier is important for maintaining a high endocochlear potential (EP) (41). We have previously shown that *Ndp*-KO mice have a reduced endocochlear potential at 1 to 3 mo (27, 28). In the current study, 3-mo-old untreated *Cdh5CreERT2;Ctnnb1^flex3^*^/+^;*WT* mice had an average EP of 124.8 mV, and untreated *Cdh5CreERT2;Ctnnb1^flex3^*^/+^;*Ndp*-KO had 91.7 mV. In treated *Cdh5CreERT2;Ctnnb1^flex3^*^/+^;*Ndp*-KO mice, average EP improved to 114.1 mV (Fig. 4*S*).

### Stabilization of β-Catenin in Vascular Endothelial Cells Prevented Cochlear Hair Cell Death in *Ndp*-KO Mice.

**Fig. 5.**
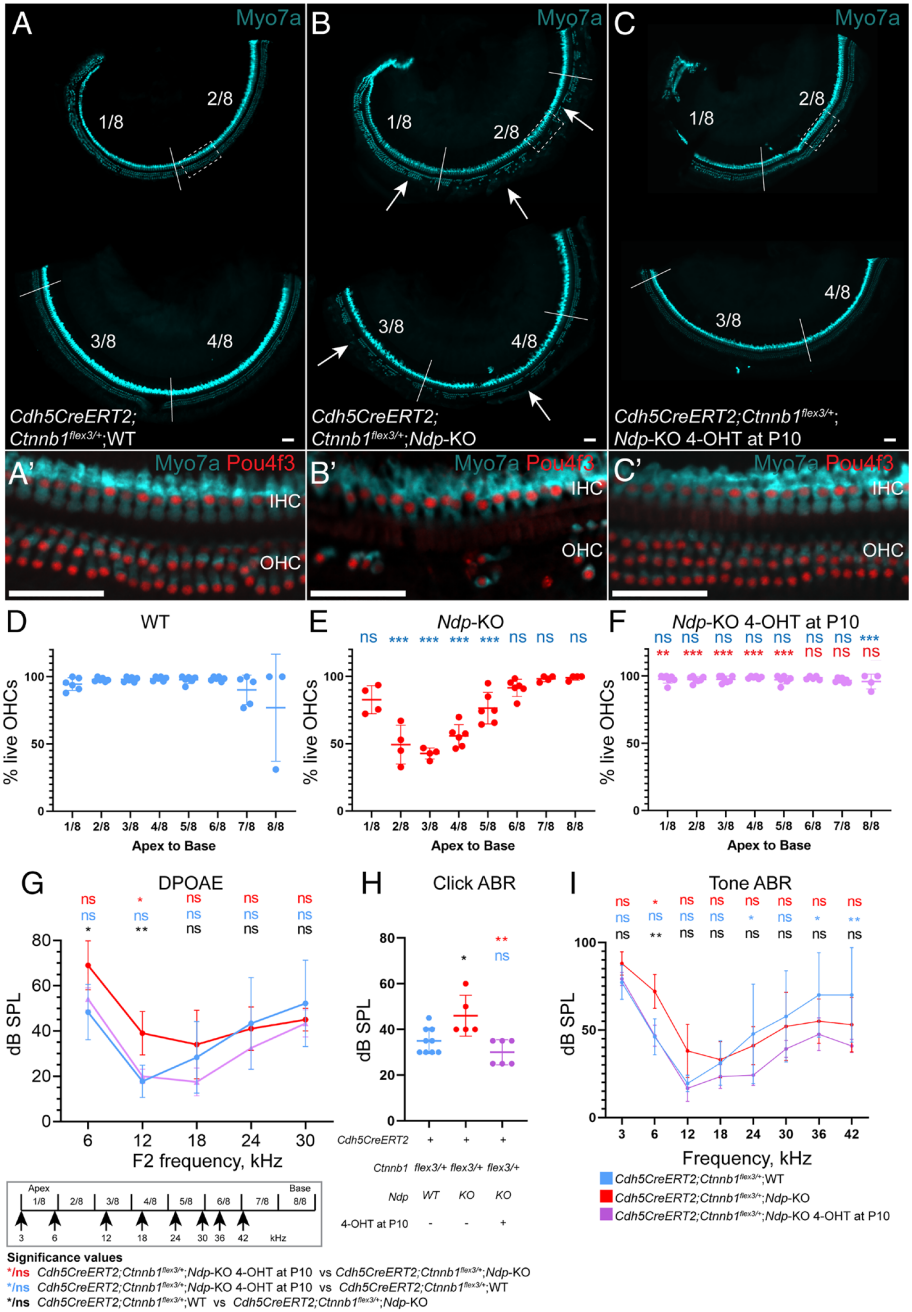
Organ of Corti whole mounts and tests of auditory function at 2 and 3 mo show OHC death and hearing loss were prevented by vascular β-catenin stabilization at P10. (*A*–*C’*) Immunostaining for Myo7a and Pou4f3 showing rescue of IHC and OHC in the organ of Corti wholemounts of treated and control mice at 2 mo. See *SI Appendix*, Fig. S10 for full-length images of the organ of Corti. (*D*–*F*) Percentage of surviving OHCs in tonotopically mapped regions of the cochlea from apex to base. (*G*–*I*) DPOAE, click ABR, and pure tone ABR threshold measurements of treated and control mice at 3 mo. Data are shown as mean ± SD; (*D*–*F*): *Cdh5CreERT2;Ctnnb1^flex3/+^;*WT n = 6, *Cdh5CreERT2;Ctnnb1^flex3/+^;Ndp*-KO n = 6, 4-OHT-treated *Cdh5CreERT2;Ctnnb1^flex3/+^;Ndp*-KO n = 6. (*G*–*I*): *Cdh5CreERT2;Ctnnb1^flex3/+^;*WT n = 9, *Cdh5CreERT2;Ctnnb1^flex3/+^;Ndp*-KO n = 5, 4-OHT-treated *Cdh5CreERT2;Ctnnb1^flex3/+^;Ndp*-KO n = 6. (*D*–*F*, *G*, and *I*) analyzed with two-way ANOVA and Tukey’s multiple comparisons test; (*H*) analyzed with one-way ANOVA and Sidak’s post hoc test; *P ≤ 0.05, **P ≤ 0.01, ***P ≤ 0.001, ****P ≤ 0.0001, and ns = nonsignificant. [Scale bar: 50 µm (*A*–*C’*).]

We used organ of Corti whole mounts stained with antibodies against the hair cell markers Myo7a and Pou4f3 (Brn3c) to assess the survival of cochlear hair cells at 2 mo (Fig. 5 and *SI Appendix*, Figs. S9 and S10). Untreated *Cdh5CreERT2;Ctnnb1^flex3^*^/+^;*WT* mice had three complete rows of OHC (Fig. 5 *A* and *A’* and *SI Appendix*, Fig. S10*A*). Untreated *Cdh5CreERT2;Ctnnb1^flex3^*^/+^;*Ndp*-KO mice had extensive loss in the mid apical region (Fig. 5 *B* and *B’* and *SI Appendix*, Fig. S10*B*). IHC loss was not observed. In treated *Cdh5CreERT2;Ctnnb1^flex3^*^/+^;*Ndp*-KO mice with vascular β-catenin stabilization, three complete rows of OHCs were preserved (Fig. 5 *C* and *C’* and *SI Appendix*, Fig. S10*C*). Treatment of *Ndp-KO* mice with 4-OHT alone in the absence of the *Cdh5CreERT2;Ctnnb1^flex3^*^/+^ alleles had no protective effect on hair cells (*SI Appendix*, Fig. S9).

Images of the organ of Corti wholemounts were mapped into eight regions of equal distance along the apex-to base axis and the percentage of surviving (Myo7a+ve) hair cells was recorded in each region. Consistent with previous observations (27, 28), untreated *Cdh5CreERT2;Ctnnb1^flex3^*^/+^;*Ndp*-KO mice had significant OHC loss in mid-apical regions 2/8 to 5/8 corresponding to 6 to 24 KHz along the tonotopic axis of the mouse cochlea. This loss was completely prevented in treated *Cdh5CreERT2;Ctnnb1^flex3^*^/+^;*Ndp*-KO mice (Fig. 5 *D*–*F*).

A group of treated and untreated mice were maintained up to 6 mo of age to assess long-term hair cell survival. At this age, analysis of lateral wall vasculature showed an even stria vascularis capillary network in untreated *Cdh5CreERT2;Ctnnb1^flex3^*^/+^;*WT* and treated *Cdh5CreERT2;Ctnnb1^flex3^*^/+^;*Ndp*-KO mice, while untreated *Cdh5CreERT2;Ctnnb1^flex3^*^/+^;*Ndp*-KO mice had extensive vessel remodeling with elongated vessel segments (*SI Appendix*, Fig. S10 *D*–*F*). Untreated *Cdh5CreERT2;Ctnnb1^flex3^*^/+^;*WT* and treated *Cdh5CreERT2;Ctnnb1^flex3^*^/+^;*Ndp*-KO mice had complete preservation of three rows of OHCs while untreated *Cdh5CreERT2; Ctnnb1^flex3^*^/+^;*Ndp*-KO mice had nearly complete OHC loss in the mid-apical region (*SI Appendix*, Fig. S10 *G*–*I’*). Some OHC degeneration was observed at the base of *Cdh5CreERT2;Ctnnb1^flex3^*^/+^; *WT* cochleas typical of C57BL/6 mice and consistent with previous observations (27, 28).

### Stabilization of β-Catenin in Vascular Endothelial Cells Preserved Auditory Function in *Ndp*-KO Mice.

OHC function was estimated at 3 mo of age by measuring distortion product otoacoustic emissions (DPOAEs) to f2 frequencies of 6 to 30 kHz. DPOAE thresholds of untreated *Cdh5CreERT2;Ctnnb1^flex3^*^/+^;*Ndp*-KO mice were significantly elevated compared to the untreated *Cdh5CreERT2;Ctnnb1^flex3^*^/+^;*WT* at 6 to 12 kHz (Fig. 5*G*, *P < 0.05, **P < 0.01, black asterisks, *SI Appendix*, Fig. S11 *A*–*C*), consistent with the observed pattern of mid-apical OHC loss (Fig. 5*E*) and with our previous report (28). The thresholds of the treated *Cdh5CreERT2;Ctnnb1^flex3^*^/+^;*Ndp*-KO mice were rescued by vascular β-catenin stabilization, they were not significantly different from untreated *Cdh5CreERT2;Ctnnb1^flex3^*^/+^;*WT* mice at these frequencies (ns, blue).

Auditory function was further evaluated by recording auditory brainstem responses (ABRs) (Fig. 5 *H* and *I*). Untreated *Cdh5CreERT2;Ctnnb1^flex3^*^/+^;*Ndp*-KO mice had significantly elevated click (broadband) ABR thresholds compared to untreated *Cdh5CreERT2;Ctnnb1^flex3^*^/+^;WT mice. Thresholds of treated *Cdh5CreERT2;Ctnnb1^flex3^*^/+^;*Ndp*-KO were rescued to a level similar to untreated *Cdh5CreERT2;Ctnnb1^flex3^*^/+^;WT (Fig. 5*H*), indicating a good level of functional hearing.

Frequency-specific ABRs showed significantly elevated thresholds in untreated *Cdh5CreERT2;Ctnnb1^flex3^*^/+^;*Ndp*-KO at 6 kHz (Fig. 5*I*, **P < 0.01, black asterisks, *SI Appendix*, Fig. S11 *D*–*F*) and a trend to increase at 3 and 12 kHz. This pattern was consistent with our previously reported observations in *Ndp*-KO mice (without the *Cdh5CreERT2* and *Ctnnb1^flex3^* alleles) at the same age (28) (*SI Appendix*, Fig. S11*H*). After 4-OHT treatment *Cdh5CreERT2;Ctnnb1^flex3^*^/+^;*Ndp*-KO showed thresholds similar to untreated *Cdh5CreERT2;Ctnnb1^flex3^*^/+^;WT mice and previously reported WT mice at these frequencies (Fig. 5*I*, ns blue, *SI Appendix*, Fig. S11*I*). This pattern of frequency-restricted hearing loss and rescue is consistent with the mid-apical pattern of OHC loss and its prevention by vascular β-catenin stabilization. Early frequency-restricted hearing loss is also observed in Norrie patients (42)

Based on these data, we propose a model of hearing loss in Norrie disease in which maintaining cochlear vasculature integrity is an essential function of norrin signaling, critical for OHC survival by providing a suitable cochlear microenvironment. Our data support the conclusion that restoring norrin signaling to endothelial cells is sufficient for normal cochlear homeostasis and function. Further, these data show that the presence of secreted norrin signaling directly to hair cells (or other cochlear cells) is not required to preserve auditory function.

### Cells of the Developing Human Cochlea Were Profiled by Single-Cell Transcriptomic Analysis.

**Fig. 6.**
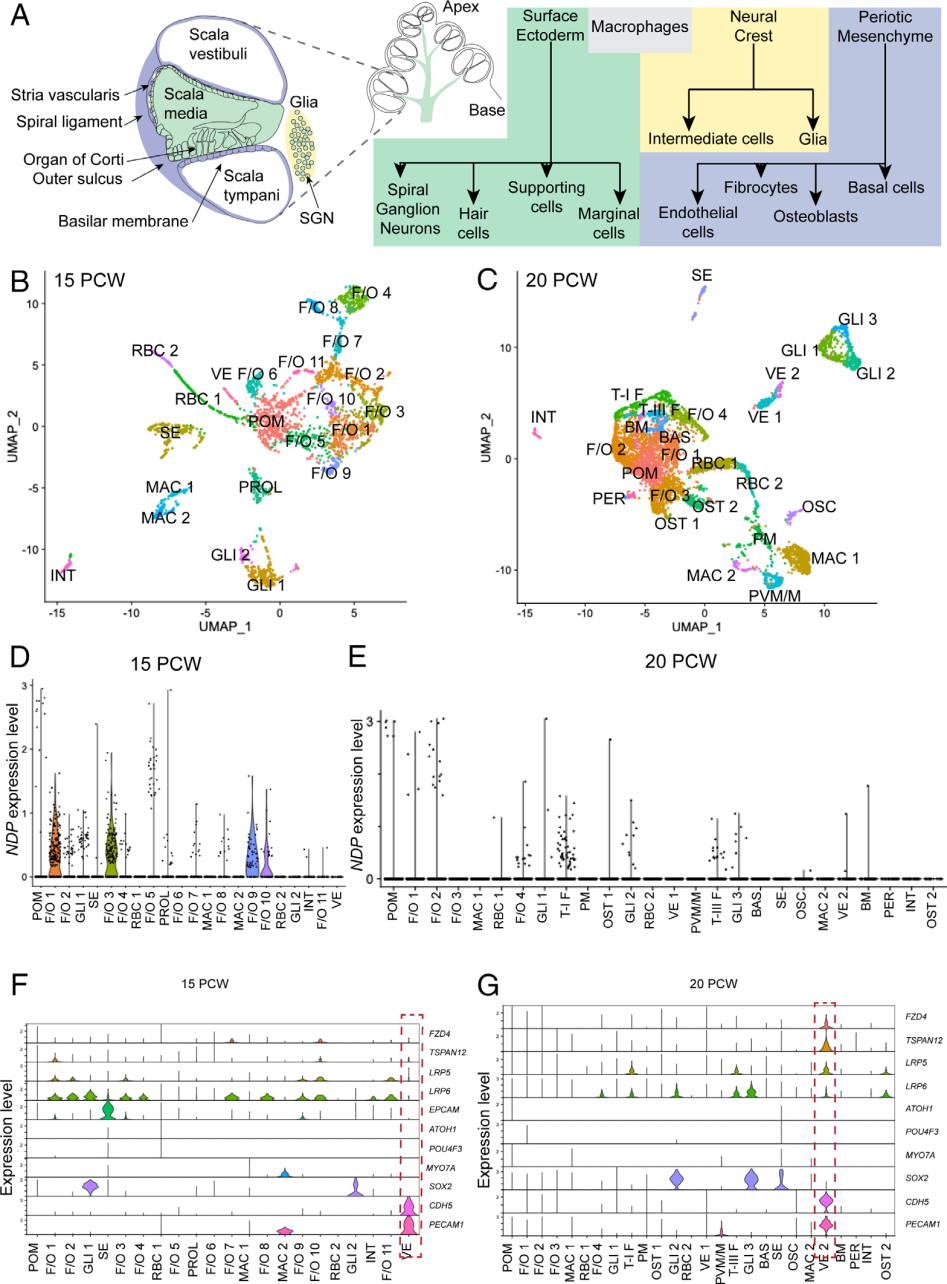
Single-cell transcriptomic analysis of the human cochlea identified *NDP*-expressing cell types and target genes consistent with vasculature being a site of direct norrin signaling. (*A*) Schematic of a mammalian cochlea showing cell types in the cochlea and their embryonic origins. Green: surface ectoderm, yellow: neural crest, purple: periotic mesenchyme, gray: unknown origin. (*B*) UMAP plot showing cell clusters identified in the 15 pcw human cochlea. (*C*) UMAP plot showing cell clusters identified in the 20 pcw human cochlea. (*D*) Violin plot showing *NDP* expression in specific cell clusters at 15 pcw. (*E*) Violin plot showing *NDP* expression in specific cell clusters at 20 pcw. (*F* and *G*) Violin plots showing expression of Norrin receptors, with sensory epithelium and endothelial cell markers in the 15 pcw (*A*) and 20 pcw (*B*) human cochlea. Dashed red boxes indicate vascular endothelial cells coexpressing Norrin receptors. POM: periotic mesenchyme, F/O: fibrocyte/osteoblast, T-I F: Type I fibrocytes, T-II F: Type III fibrocytes (spiral ligament fibrocytes), BM: basilar membrane, BAS: basal cells, INT: intermediate cells, VE: vascular endothelial cells, PER: pericytes, SE: sensory epithelium, GLI: glia, OST: osteoblasts, OSC: osteoclasts, MAC: macrophages, PM: proliferating macrophages, PVM/M: perivascular macrophage-like-melanocytes.

To determine whether the disease mechanism, elucidated from a mouse model, is consistent with expression of *NDP*, and norrin receptor and target genes, in the human cochlea, we analyzed the developing human cochlea at 15 to 20 postconception weeks (pcw) during the period of vascular development. As very limited data exist on the developing human cochlear transcriptome, we first profiled cochlear cell populations using the 10× Genomics platform. The mammalian cochlea is derived from multiple embryonic tissue sources (Fig. 6*A*). The otocyst arising from the surface ectoderm forms the walls of the *scala media*, including the sensory epithelium (Fig. 6*A*, green) (43, 44). The periotic mesenchyme forms the walls of the *scala tympani* and *scala vestibuli* (45) (Fig. 6*A*, purple). Neural crest cells contribute the intermediate cells/melanocytes of the *stria vascularis* and glia of the spiral ganglion (Fig. 6*A*, yellow) (46–48). The cochlea also contains resident macrophages of unclear origin, associated with vasculature (Fig. 5*A*, gray) (49).

From the 15 pcw cochlea (17 gestational weeks) (Fig. 6*B*), 22 clusters of cells were annotated based on known cell-type markers from published literature (Dataset S1). Clusters identified consisted of glia (GLI1-2), sensory epithelial cells, (SE) (*SI Appendix*, Fig. S12*A*) intermediate cells/melanocytes (INT), macrophages (MAC1-2), vascular endothelium (VE), and red blood cells (RBC1-2) (Fig. 6*B* and *SI Appendix*, Fig. S12 *A* and *B*). SE cells expressing *S100A1* and *FBXO2* were identified as hair and supporting cell precursors. No mature hair cells expressing *POU4F3*, *MYO7A,* and *ATOH1* were identified at this stage. The remaining clusters expressing *POU3F4* (*SI Appendix*, Fig. S12*C*) (50) were of the fibrocyte/osteoblast (F/O) lineage derived from the periotic mesenchyme (POM). One *OMD*+ve osteoblast cluster was identified. (*SI Appendix*, Fig. S12*C*).

At 20 pcw (22 gw), when a more mature *stria vascularis* and organ of Corti are expected (51, 52) (*SI Appendix*, Fig. S13*A*), 26 clusters of cells (Dataset S2) were annotated (Fig. 6 *C* and *F* and *SI Appendix*, Fig. S13 *B*–*F*). Glial cells had resolved into three clusters (GLI1-3) all expressing *MBP* and *MPZ*, while two (GLI 2 and GLI 3) also expressed *SOX2*, *SOX10,* and *FOXD3* (*SI Appendix*, Fig. S13*B*). Some SE cells now expressed the supporting cell marker *SOX2*. Only four sensory hair cells (with >0 expression level for *ATOH1*, *MYO7A,* or *POU4F3*) were identified. Fibrocyte subtypes had emerged (*SI Appendix*, Fig. S13 *D* and *E*) including Type I fibrocytes (T-I F), Type III fibrocytes of the spiral ligament (T-III F), precursors of basal cells (BAS), and cells of the basilar membrane (BM). The remaining fibrocyte clusters were labeled F/O 1 to 4. One of these expressed *TNC* encoding an extracellular matrix protein of the basilar membrane and spiral lamina (F/O 4). Two clusters of osteoblasts (OST 1 and OST 2) were present.

Two vascular endothelial clusters (VE1-2) and one pericyte cluster (PER) were identified. VE1 expressed *SOX18*, *CAV1,* and *EGFL7* (53–55); VE2 also expressed *CDH5* and *PECAM1* (56, 57) (*SI Appendix*, Fig. S13*F*). Of four clusters of macrophages (MAC), one was identified as perivascular macrophage-like-melanocytes of the stria (PVM/M) and another as osteoclasts (OSC). A final group expressed proliferation markers (PM). No spiral ganglion neurons or marginal cells were detected.

### Expression of NDP and the Norrin Receptor Complex in the Human Cochlea Is Consistent with a Vascular Norrie Disease Mechanism.

At 15 pcw, *NDP* was widely expressed in F/O 1, 3, 9, 10 clusters and a small proportion of cells in F/O 2, 4, 5, 7, 8 and in POM and GLI 1 (Fig. 6*D* and *SI Appendix*, Fig. S14*A*). At 20 pcw, expression was more restricted. *NDP* +ve cells were identified in TIII-F (*SLCO1C1* +ve) and TI-F (low *CLDN11*+ve) fibrocyte clusters and F/O 4, likely from the basilar membrane or spiral lamina (Fig. 6*E* and *SI Appendix*, Fig. S14*B*). Our previous analyses of the adult mouse cochlea (28, 58) showed *Ndp* expression in basal cells. In contrast, no *NDP* expression was observed in BAS in the 20 pcw human cochlea. However, basal cells are not completely differentiated from surrounding fibrocytes at this stage (59). Alternatively, *NDP* expression in basal cells may have a later onset. *NDP* was also expressed in GLI 2 and GLI3. Within *NDP*+ve clusters only a fraction of cells expressed *NDP*, which may reflect a naturally low expression level and/or a degree of gene dropout known to occur in scRNA datasets (60). No expression was detected in the SE, VE, PER, or MAC clusters.

At 15 pcw, all norrin receptors (*FZD4, TSPAN12, LRP5,* and *LRP6*) showed a low-level expression in multiple cell types (Fig. 6*F*), including the VE cluster. By 20 pcw expression of all four receptors coincided in one cluster, VE2, consistent with these cells being targets of Norrin signaling (Fig. 6*G*). VE1 did not express these receptors. Targets of Norrin signaling (*CLDN5, ABCB1 SOX17, SLC7A1,* and *SLC7A5*), previously identified and temporally analyzed in the mouse cochlea (27, 28), were expressed in the VE at 15 pcw (*SI Appendix*, Fig. S14 *C* and *D*). At 20 pcw they were expressed in VE2 which also coexpressed Norrin receptors. Only *CLDN5* was expressed, at a lower level, in VE1, which did not express Norrin receptors. *SLC7A5* was also expressed in the INT and T-III F clusters, suggesting that it may play a role in transport of amino acids to supply the stria vascularis, a highly metabolically active structure (61).

Alternative norrin receptor genes *LGR4, 5,* and *6* were not expressed in VE or SE cells. At 15 pcw, *LGR4* had a relatively widespread expression in F/O 1-2, 7-8, 9, and 11 and GLI1. By 20 pcw, this expression was present in T-III F, F/O4, and OST2 only (*SI Appendix*, Fig. S15). Expression of norrin receptor genes in endothelial cells was consistent with that in the mouse (*SI Appendix*, Fig. S16).

## Discussion

### Hair Cell Death in the Cochlea Is Secondary to Vascular Defects.

Our previous work has shown that morphological changes in lateral wall vasculature and a breakdown of the vascular barrier precede the death of OHCs (27) in the *Ndp*-KO mouse. Genes specific to endothelial cells were shown to be dysregulated in the absence of norrin (28) (Fig. 3 and *SI Appendix*, Figs. S6 and S7). In the current study, we provide evidence supporting a causal relationship between the early vascular phenotype and later OHC death and hearing loss. Restoration of vascular integrity was sufficient to prevent OHC death in *Ndp*-KO mice, in the absence of secreted norrin. As previously demonstrated in the retina (6) we also show that norrin likely does not provide directional cues to or exert gradient effects on cochlear vasculature as the phenotype could be rescued by an intervention intrinsic to endothelial cells.

We used an endothelial cell–specific (32) inducible Cre line (Chd5CreERT2) to drive β-catenin stabilization in *Ndp*-KO mice. Endothelial specificity of this system was verified in the cochlear vasculature as indicated by our observations using anti-β-catenin and Estrogen receptor immunostaining and using the Ai9 recombination reporter as a proxy. The Ai9 allele (34) and other reporters in the ROSA26 locus have been used to report hair cell-specific Cre driver activity (e.g., *Atoh1*-Cre, *Myo15*-Cre, or *Prestin*-CreERT2 induced at timepoints from P2 to P22) and show fluorescent signal in hair cells (62–64). It did not report Cre activity in hair cells with the Cdh5CreERT2 driver in our study. We found the Cdh5CreERT2 driver was not completely silent in the uninduced state (i.e., without tamoxifen) as we did detect some uninduced Cre activity. However, this was rare, and when observed, it was only in a fraction of endothelial cells (*SI Appendix*, Figs. S4 and S5) and not sufficient to prevent vascular morphological and barrier defects (Fig. 4 *G*–*I*), hair cell death (Fig. 5 *B* and *E* and *SI Appendix*, Fig. S10 *B*, *E*, and *H*), or hearing loss (Fig. 5 *G*–*I* and *SI Appendix*, Fig. S11 *H* and *K*) in the absence of Cre induction. Uninduced *Cdh5CreERT2; Ctnnb1flex3/+; Ndp*-KO mice had elevated hearing thresholds similar to previously reported *Ndp*-KO mice (*SI Appendix*, Fig. S11*H*). The phenotype was rescued only on Cre induction by 4-OHT administration, driving recombination in all endothelial cells.

It has previously been suggested that norrin binds directly to Fzd4 on OHCs regulating OHC maturation and survival with the absence of norrin resulting in early downregulation of β-catenin targets in hair cells (26). We found that this interaction may not be essential for hair cell survival, provided the vascular barrier is maintained. The same study also showed that early genetic stabilization of β-catenin in hair cells promoted hair cell survival in the absence of norrin signaling. However, β-catenin stabilization has been shown to play an antiapoptotic role in hair cells and spiral ganglion neurons against forms of environmental stress (65, 66). Direct β-catenin stabilization in OHCs may be playing a similar protective role against the stress of an abnormal environment in the *Ndp*-KO cochlea, potentially unrelated to the activity of native norrin. Nevertheless, considering the studies together indicates that OHCs and hearing can be protected either by β-catenin activation in endothelial cells (this study) or alternatively in hair cells (26). As different *Ndp-KO* lines were used in these studies, genetic background effects could influence the disease presentation, although both were loss of function mutations. Our data strongly suggest that repair of the vascular barrier defects will be essential for long-term preservation of hearing.

We observed hair cell survival up to 6 mo after β-catenin stabilization in endothelial cells, although 3 mo was the oldest timepoint analyzed using DPOAE and ABR. The *Ndp-KO* model is maintained on a C57BL6 background. The presence of the *Cdh23^ahl^* allele in the BL6 strain confers increased susceptibility to age-related hearing loss in the high-frequency region at ages beyond 3 mo (67) confounding the assessment of *Ndp*-KO-related hearing loss at older ages in this background. Indeed, variability in ABR thresholds was found between individual mice tested in this study at high frequencies (*SI Appendix*, Fig. S11 shows plots of individual mice) and in our previous reports (28).

### Endocochlear Potential Reduction and Dysregulation of Multiple Functions of the Cochlear Vasculature in the Norrie Disease Cochlea May Contribute to Stress on OHCs.

The separation of the three fluid-filled compartments of the cochlea by cellular epithelia and the vascular endothelial barrier enables the efficient recycling of K^+^ ions through the stria vascularis into the endolymph, generating a high endocochlear potential (EP) (45). *Ndp*-KO mice have a mildly reduced EP of 90 to 100 mV [Fig. 4*S* and (27)]. OHC are thought to be tolerant of EPs as low as 18 to 30 mV for 1 to 3 mo or longer (68, 69) based on studies of mouse models with defects in specific strial cell types. However, mildly reduced EP in *Ndp*-KO mice may cause long-term stress and affect hair cell function (68, 70, 71). Increased permeability of vessels in the *Ndp*-KO mouse likely affects ionic content of the endolymph, in addition to the composition of the perilymph and intrastrial fluid involving molecules other than K+.

Vascular barrier abnormalities have been implicated in hearing loss (41, 72, 73). Two major pathways of vessel permeability (74) are affected in the *Ndp*-KO. Expression of *Cldn5* (regulating paracellular permeability) and *Plvap* (regulating transcellular permeability) was abnormal (Fig. 3 and *SI Appendix*, Fig. S2) (27, 28) and restored by vascular β-catenin stabilization. Barrier endothelial cells also express efflux transporters to eliminate neurotoxic substances (75). For example, Abcb1a, a target of norrin signaling (Fig. 3 and *SI Appendix*, Fig. S6) (27, 28) effluxes hydrophobic metabolites and/or drugs into the blood stream (76) preventing ototoxicity (77). The endothelial transporters *Slc7a1* and *Slc7a5* (37, 78) are also regulated by norrin signaling (Fig. 3 and *SI Appendix*, Fig. S6) (27, 28). *SLC7A5* was also expressed in intermediate cells in the 15 pcw and 20 pcw human cochlea (Fig. 6). Both are involved in the uptake of amino acids at the blood–brain barrier (79, 80) and their reduced expression in the cochlea may result in insufficiency. Membrane transporters deliver critical nutrients in the compartmentalized cochlea (36, 81). Hence, we suggest that it is the dysregulation of these multiple functions of the cochlear vascular barrier in the *Ndp*-KO that cause an abnormal intracochlear environment, metabolic insufficiency, reduced endocochlear potential leading to hair cell death and hearing loss over a period of time.

### Identification of Downstream Targets of Norrin Signaling.

Identification of the vascular endothelial genes regulated by the β-catenin pathway may give important insights for the development of pharmacological therapies. Of the multiple vascular endothelial cell genes regulated by β-catenin, *Cldn5* showed markedly consistent downregulation in the *Ndp*-KO cochlea and responsiveness to β-catenin stabilization. Cldn5 is a major tight junction protein in brain and retinal vasculature (82, 83), a reduction leads to a disruption of barrier function and is associated with many neurological diseases (84). These observations suggest that reduction of *Cldn5* expression in the *Ndp*-KO cochlea may be a critical factor leading to OHC death and increasing Cldn5 expression in all endothelial cells may be sufficient to prevent hearing loss in Norrie disease.

Some of the genes analyzed (*Abcb1a*, *Slc7a1*, and *Slc7a5*) did not reach significance between the untreated *Cdh5CreERT2; Ctnnb1^flex3^*^/+^*;WT* and *Cdh5CreERT2; Ctnnb1^flex3^*^/+^*; Ndp-KO* at 2 mo (Fig. 3 *H*–*J*, blue versus red) although they did at 1 mo (Fig. 3 *B*–*D*) and responded to β-catenin stabilization. Lack of significance at the later timepoint could be due to the presence of one Cre allele, which showed rare, endothelial-specific activity (*SI Appendix*, Figs. S4 and S5) without 4-OHT, although not enough to prevent vascular barrier defects and hair cell death.

Single-cell transcriptomic analysis of the developing human cochlea confirmed that vascular endothelial cells are likely targets of secreted norrin. Of the 26 cell-type clusters identified in the human scRNA dataset, the expression of norrin receptors coincided in only vascular endothelial cells. However, some cell types were missing from these data, such as marginal cells. Also, there were very few (~4 to 5) hair cells. Therefore, we cannot completely exclude expression in these cell types, especially if the expression is low

### Implications for the Treatment and Management of Norrie Deafness.

These observations will inform the development of a precisely targeted, clinically applicable therapy for Norrie disease. In recent work, we have shown that the phenotype is responsive to norrin supplied as an AAV-mediated gene therapy (28). Most gene therapies for hearing loss have been targeted to cochlear hair cells (85), with the vector delivered directly to the cochlea or being capable of crossing the vascular barrier if administered systemically. A therapy targeted to vascular endothelial cells could be delivered systemically without needing to cross the barrier. Wnt surrogate antibodies delivered systemically have been used to treat brain and retinal vasculature (86, 87). Pharmacologically increasing β-catenin signaling in vascular endothelial cells to levels sufficient for barrier maintenance may be sufficient to prevent hearing loss in Norrie disease.

We noted that *Cldn5*, *Abcb1a*, and *Sox17* were expressed at the highest level in treated *Cdh5CreERT2;Ctnnb1^flex3/+^;WT mice*, consistent with dose-dependent response to levels of stabilized β-catenin (with a trend of untreated *Cdh5CreERT2;Ctnnb1^flex3/+^; Ndp*-KO, < untreated *Cdh5CreERT2;Ctnnb1^flex3^*^/+^; WT < treated *Cdh5CreERT2;Ctnnb1^flex3/+^;* WT). We cannot exclude the possibility that norrin-independent β-catenin responsive genes, not included in our Norrie biomarker panel, or other interacting pathways were affected by artificially high β-catenin levels; however, we did not find obvious adverse effects upon histological examination (*SI Appendix*, Fig. S8).

Precisely controlling timing and levels of β-catenin signaling may be key to restoring barrier function without causing adverse effects. It was reported that overactivation of β-catenin signaling in vascular endothelial cells, either due to mutations in *Ctnna1/CTNNA1* or the norrin receptor Lrp5/LRP5 in humans and mice, or using the *Ctnnb1^flex3^* mouse allele, led to defects in the retinal vasculature resembling FEVR (88). The most severe vascular phenotype was seen in *Ctnnb1^flex3/flex3^* homozygous mice, carrying the WT *Ndp* allele and induced at P3, resulting in very high levels of stabilized β-catenin (200 fold above WT). In contrast, we did not observe adverse effects in our *Cdh5CreERT2;Ctnnb1^flex3/+^;Ndp*-KO mice induced at the later timepoint P10, and containing a single *Ctnnb1^flex3^ allele*.

In the short term, a better molecular-level understanding of the Norrie disease cochlear vascular phenotype may help improve clinical management of patients. For example, mice lacking functional *Abcb1a* show increased sensitivity to ototoxicity (77). We show that *Ndp*-KO mice have reduced levels of *Abcb1a* in the cochlea (Fig. 2) (28). Identification of substrates of the human orthologue *ABCB1* could help minimize ototoxicity in individuals with Norrie disease.

## Conclusion

The findings from this study support a Norrie disease mechanism in the cochlea whereby maintaining vasculature integrity and a suitable cochlear microenvironment is an essential function of norrin signaling, critical for OHC survival. We show that norrin signaling to hair cells or other cell types is not required to preserve auditory function, provided that the vascular barrier is intact and the intracellular effector of norrin signaling to endothelial cells is restored. Finally, a better understanding of the role of cochlear microvasculature in Norrie disease may give important insights into other forms of deafness, including age-related deafness, Meniere’s disease, or Alport syndrome which may have a vascular origin.

## Methods

All procedures were performed in accordance with the UK Animals Act 1986 under UK Home Office license. The following mouse alleles *Ndp^tm1Wbrg^*; *Cdh5CreERT2^tg/tg^*, *Ctnnb1^tm1Mmt^* (referred to as *Ctnnb1^flex3^*) and Ai9 Cre reporter were crossed in a C57BL/6J background. Cochlea and retina were analyzed at 1, 2, and 6 mo by immunohistochemistry, fluorescent tracer assays, and qRT-PCR. Auditory electrophysiological testing was performed at 3 mo. Human fetal cochlea samples provided by the Human Developmental Biology Resource with ethics approval were analyzed by 10× single-cell RNA sequencing and the Seurat package. For all other methodological details, please see *SI Appendix*. Datasets produced in this study are available in Array Express E-MTAB-14063.

## Supplementary Material

Appendix 01 (PDF)

Dataset S01 (XLSX)

Dataset S02 (XLSX)

## Data Availability

Single Cell RNASeq data have been deposited in ArrayExpress (E-MTAB-14063) (89). All other data are included in the article and/or supporting information.
