# Supplementary material for: Rescue of cochlear vascular pathology prevents sensory hair cell loss in Norrie disease
Source: Proc Natl Acad Sci U S A. 2024 Nov 25;121(49):e2322124121. doi: 10.1073/pnas.2322124121 (PMC11626139; doi:10.1073/pnas.2322124121)
Supplement: Supplementary file 1 — Appendix 01 (PDF) [file pnas.2322124121.sapp.pdf]

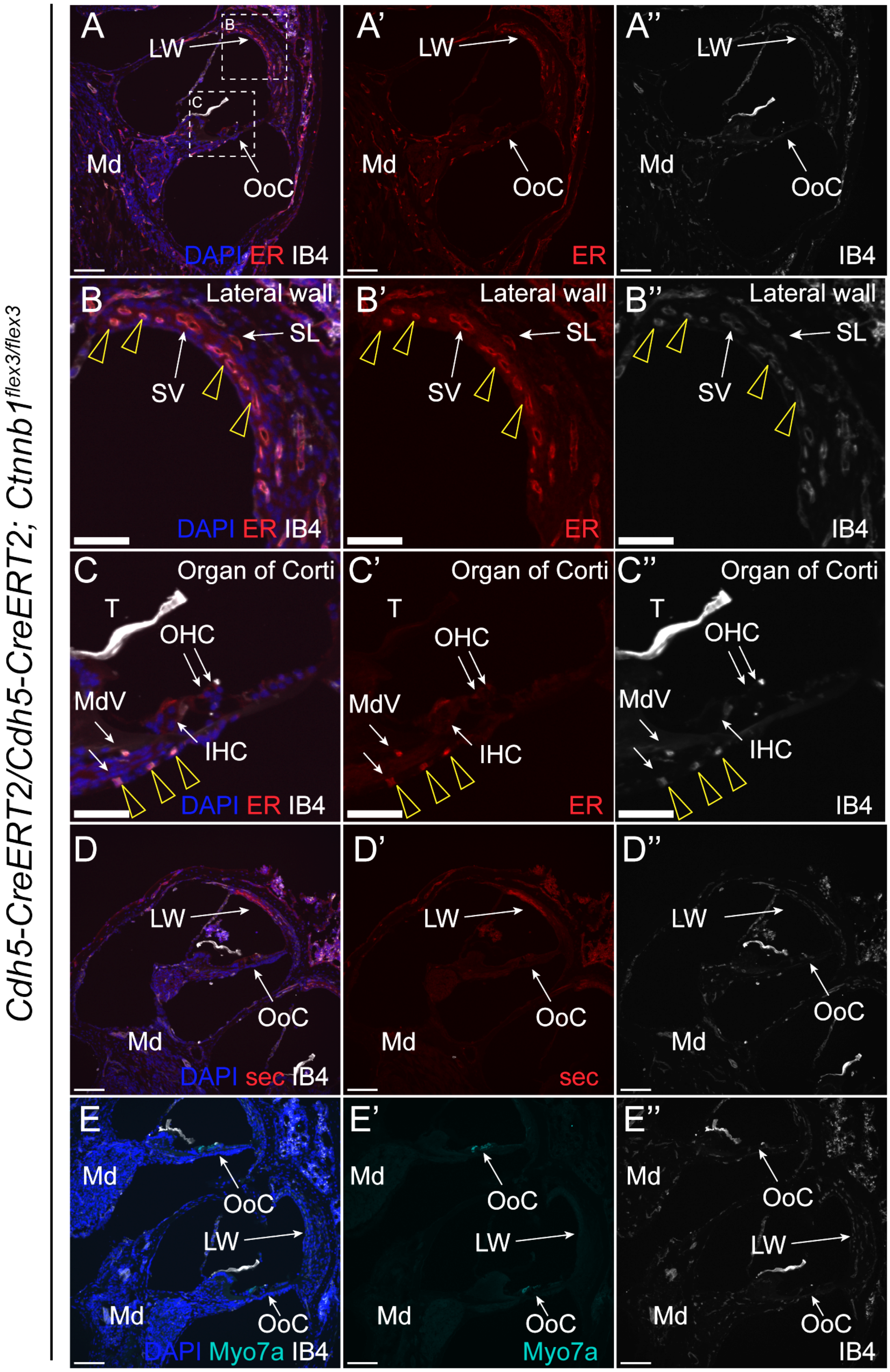
Cdh5-CreERT2/Cdh5-CreERT2; Ctnnb1 flex3/flex3
A
LW
B
C
Md
OoC
DAPI ER IB4
A'
LW
Md
OoC
ER
A''
LW
Md
OoC
IB4
B
Lateral wall
SL
SV
DAPI ER IB4
B'
Lateral wall
SL
SV
ER
B''
Lateral wall
SL
SV
IB4
C
Organ of Corti
T
OHC
MdV
IHC
DAPI ER IB4
C'
Organ of Corti
T
OHC
MdV
IHC
ER
C''
Organ of Corti
T
OHC
MdV
IHC
IB4
D
LW
OoC
Md
DAPI sec IB4
D'
LW
OoC
Md
sec
D''
LW
OoC
Md
E
Md
OoC
LW
Md
OoC
DAPI Myo7a IB4
E'
Md
OoC
LW
Md
OoC
Myo7a
E''
Md
OoC
LW
Md
OoC
IB4

Supplementary Figure 1: Specific expression of Cre-ERT2 in vascular endothelial cells.

A-C': Immunostaining for Cre-ERT2 enzyme using anti-Estrogen Receptor antibody (ER, red), on cryosections through the cochlea of Postnatal day (P) 10, *Cdh5CreERT2/Cdh5CreERT2;Ctnnb1*$^{flex3/flex3}$ mice. Vessels were counterstained with fluorescently labelled IsolectinB4 (IB4, white) which binds to endothelial cells. B-B' and C-C' show boxed regions from A. Specific ER staining (yellow arrowheads) was observed in vascular endothelial cells of the lateral wall (LW), including the stria vascularis (SV) and spiral ligament (SL) capillaries and modiolar vasculature (MdV). It was not detected in Inner (IHC) or outer hair cells (OHC).
Note, IB4 also stains the tectorial membrane (T).
D-D'': Immunostaining controls with secondary antibodies showed only non-specific background fluorescence in multiple cell types in the *Cdh5CreERT2/Cdh5CreERT2;Ctnnb1*$^{flex3/flex3}$ cochlea.
E-E'': Immunostaining with an anti-Myosin7A antibody in a cryosection parallel to A.

Cdh5CreERT2/Cdh5CreERT2;Ctnnb1$^{flex3/flex3}$ n= 3.
Scale bars: 100 µm (A-A'', D-D'',E-E'',) 50 µm (B-B'', C-C'',).

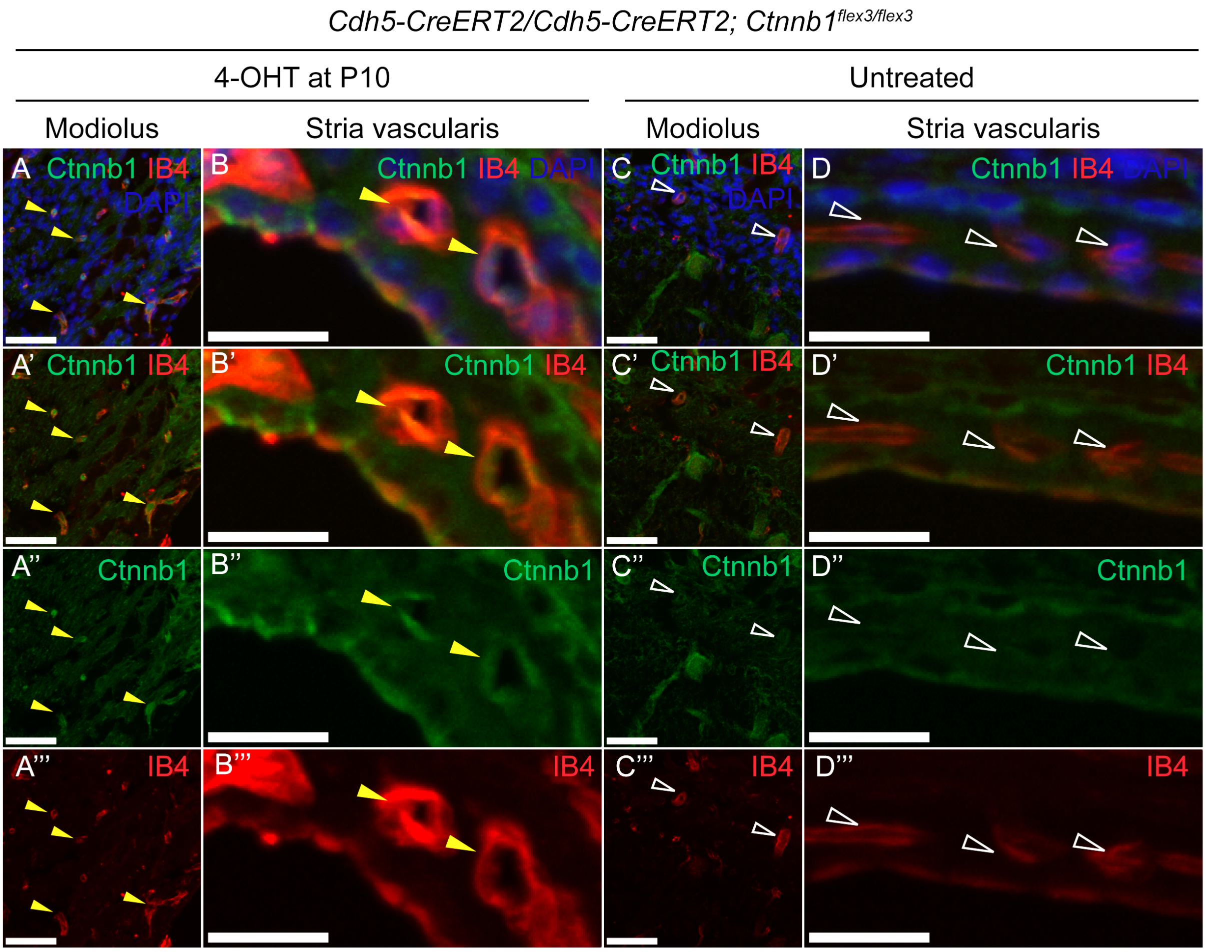
Cdh5-CreERT2/Cdh5-CreERT2; Ctnnb1flex3/flex3
4-OHT at P10
Untreated
Modiolus
Stria vascularis
Modiolus
Stria vascularis
A Ctnnb1 IB4 DAPI
B Ctnnb1 IB4 DAPI
C Ctnnb1 IB4 DAPI
D Ctnnb1 IB4 DAPI
A’ Ctnnb1 IB4
B’ Ctnnb1 IB4
C’ Ctnnb1 IB4
D’ Ctnnb1 IB4
A’’ Ctnnb1
B’’ Ctnnb1
C’’ Ctnnb1
D’’ Ctnnb1
A’’’ IB4
B’’’ IB4
C’’’ IB4
D’’’ IB4

Supplementary Figure 2: Cytoplasmic accumulation of stabilized β-catenin in vascular endothelial cells in the cochlea of *Cdh5-CreERT2/Cdh5-CreERT2; Ctnnb1$^{flex3/flex3}$* mice following 4-OHT treatment.

A-B''': Anti- β-catenin immunostaining (green) in the modiolus (A-A''') and lateral wall (B-B''') of treated *Cdh5-CreERT2/Cdh5-CreERT2; Ctnnb1$^{flex3/flex3}$* mice at 3 weeks of age after 4-OHT treatment at P10. Cytoplasmic β-catenin accumulation was observed in vascular endothelial cells (yellow arrowheads) counterstained with Isolectin B4 (IB4, red).
C-D''': Anti- β-catenin immunostaining (green) in the modiolus (C-C''') and lateral walls (D-D''')of untreated control *Cdh5-CreERT2/Cdh5-CreERT2; Ctnnb1$^{flex3/flex3}$* mice at 3 weeks of age. No β-catenin accumulation was observed in vascular endothelial cells (white arrowheads) counterstained with Isolectin B4 (IB4, red).
Note: As expected, background levels of β-catenin were detected in multiple cell types.

Images are tile scans of images acquired on a spinning disk confocal microscope (Nikon Eclipse Ti2, Crest Optics).

4-OHT treated *Cdh5-CreERT2/Cdh5-CreERT2; Ctnnb1$^{flex3/flex3}$* n= 4, untreated *Cdh5-CreERT2/Cdh5-CreERT2; Ctnnb1$^{flex3/flex3}$* n = 4. Scale bars: 50 µm (A-A''', C-C'''), 20 µm (B-B''', D-D''').

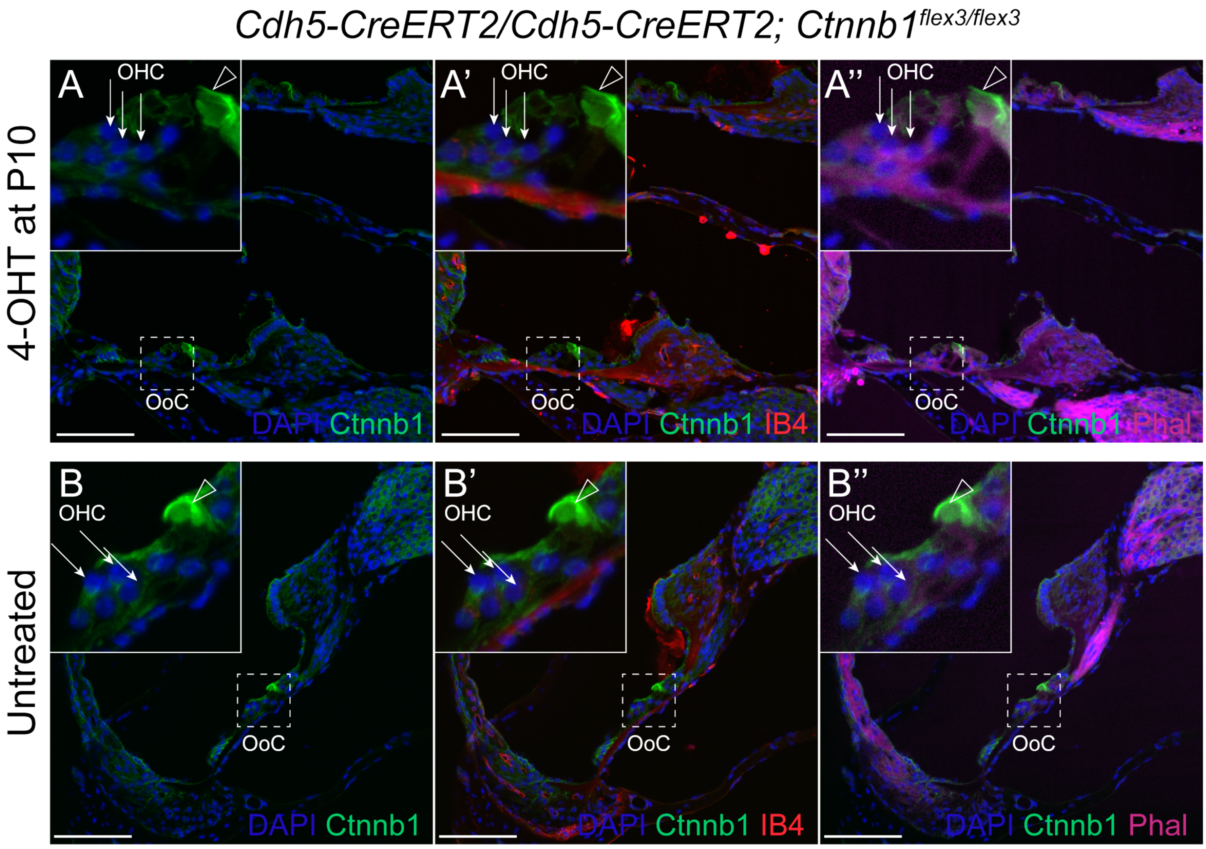
Cdh5-CreERT2/Cdh5-CreERT2; Ctnnb1flex3/flex3
4-OHT at P10
Untreated
A
A'
A''
B
B'
B''
OHC
OoC
DAPI Ctnnb1
DAPI Ctnnb1 IB4
DAPI Ctnnb1 Phal

Supplementary Figure 3: Accumulation of stabilized β-catenin is not observed in outer hair cells of *Cdh5-CreERT2/Cdh5-CreERT2; Ctnnb1$^{flex3/flex3}$* mice following 4-OHT treatment.

A-B'': Anti-β-catenin staining (green) in the organ of Corti (OoC) of treated *Cdh5-CreERT2/Cdh5-CreERT2; Ctnnb1$^{flex3/flex3}$* mice (A-A'') and untreated age-matched controls (B-B''). Insets show magnified views of the boxed region in each panel. Counter staining with IB4 (red, indicating blood vessels) and phalloidin (magenta, binding to actin filaments) shows the structure of the cochlea. Cytoplasmic β-catenin accumulation was not observed in outer hair cells (OHC, arrows) in treated mice at levels higher than untreated control mice (C-C', OHC, arrows). Note: Strong anti- β-catenin staining was also observed in some supporting cells in both the treated and untreated control mice (open arrowheads).
Images are tile scans of images acquired on a spinning disk confocal microscope (Nikon Eclipse Ti2, Crest Optics).

4-OHT treated *Cdh5-CreERT2/Cdh5-CreERT2; Ctnnb1$^{flex3/flex3}$* n= 4, untreated *Cdh5-CreERT2/Cdh5-CreERT2; Ctnnb1$^{flex3/flex3}$* n = 4, Scale bars: 100 μm.

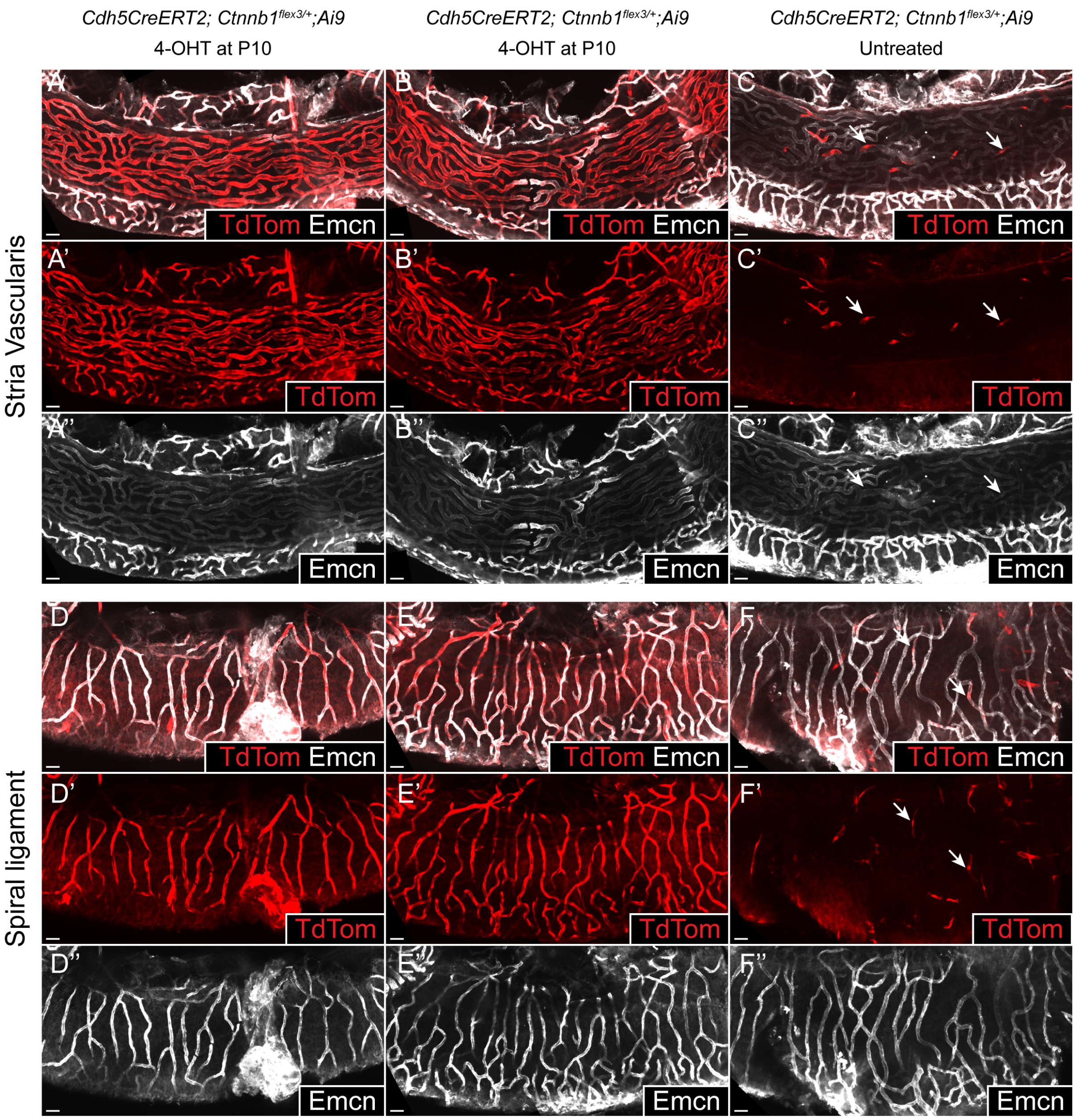

Cdh5CreERT2; Ctnnb1flex3/+;Ai9
4-OHT at P10
Cdh5CreERT2; Ctnnb1flex3/+;Ai9
4-OHT at P10
Cdh5CreERT2; Ctnnb1flex3/+;Ai9
Untreated
Stria Vascularis
Spiral ligament
A
B
C
A'
B'
C'
A''
B''
C''
D
E
F
D'
E'
F'
D''
E''
F''
TdTom Emcn
TdTom
Emcn

Supplementary Figure 4: TdTomato localization in endothelial cells of the cochlear lateral wall of treated *Cdh5-CreERT2; Ctnnb1$^{flex/+}$; Ai9* mice.

A-F': Immunostaining for TdTomato in lateral wall wholemounts of *Cdh5-CreERT2; Ctnnb1$^{flex/+}$; Ai9* mice at 1 month after 4-OHT treatment at P10. Following 4-OHT treatment, Cre activity in endothelial cells recombined the Ai9 Cre reporter allele to produce the fluorescent protein TdTomato (TdTom, red) in vessels of the stria vascularis (A-B'') and spiral ligament (D-E''). Endothelial cells were counterstained with an anti-endomucin antibody (Emcn, white). Rarely, evidence of Cre activity was observed in vascular endothelial cells in the absence of 4-OHT induction (C-C'', F-F'', arrows). We observed 10-20 tdTomato + ve cells per field of view of the lateral wall (5 mm x 2.5 mm) with uninduced Cre activity. A-F'' show tile scans of single plane images acquired on a spinning disk confocal microscope (Yokogawa, CSU22).

4-OHT treated *Cdh5-CreERT2; Ctnnb1$^{flex/+}$; Ai9* n= 4, untreated *Cdh5-CreERT2; Ctnnb1$^{flex/+}$; Ai9* n = 3. Scale bars: 200 µm.

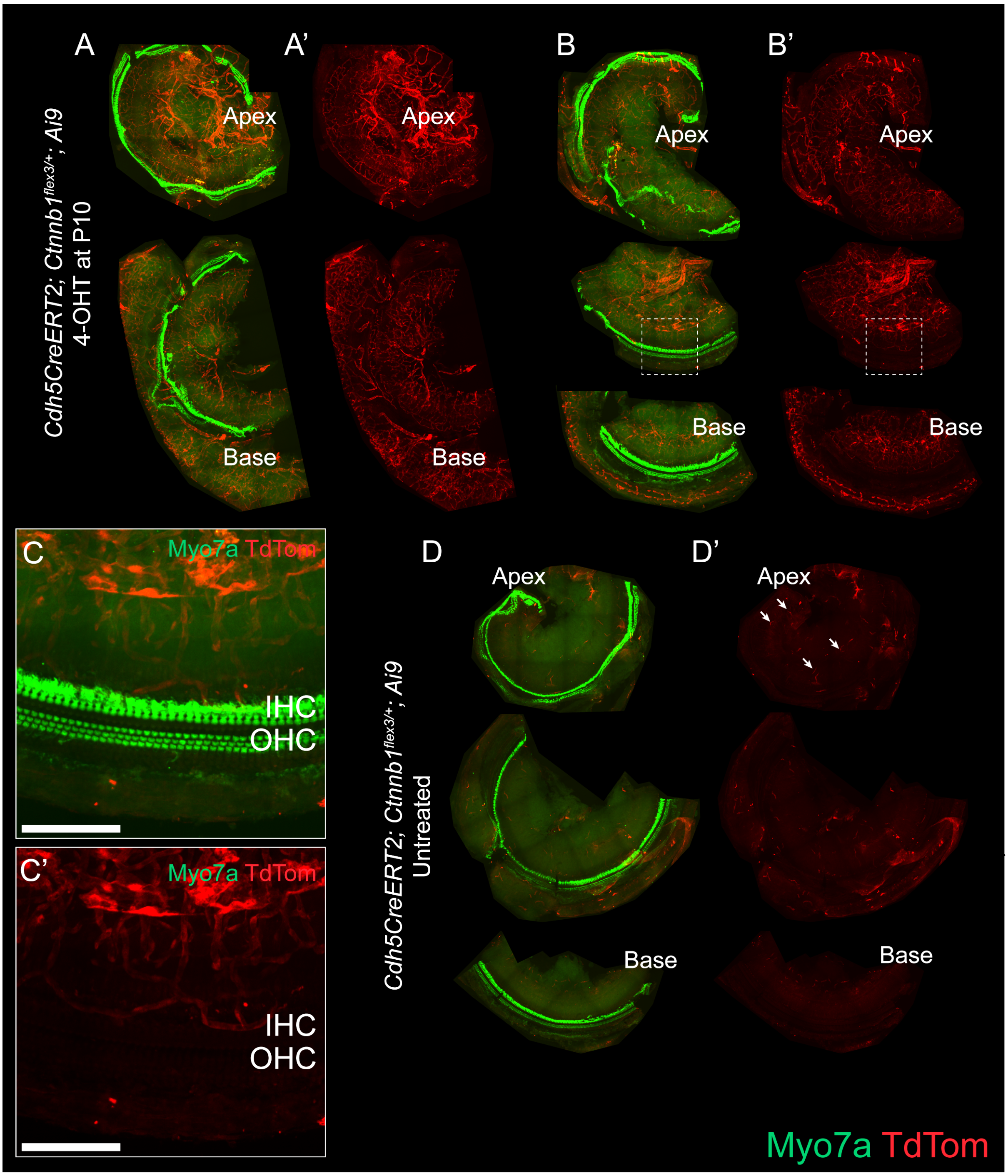
A
A'
B
B'
Cdh5CreERT2; Ctnnb1flex3/+; Ai9
4-OHT at P10
Apex
Apex
Apex
Apex
Base
Base
Base
Base
C
Myo7a TdTom
IHC
OHC
C'
Myo7a TdTom
IHC
OHC
D
D'
Cdh5CreERT2; Ctnnb1flex3/+; Ai9
Untreated
Apex
Apex
Base
Base
Myo7a TdTom

Supplementary Figure 5: TdTomato localization in the modiolus of *Cdh5-CreERT2; Ctnnb1$^{flex/+}$; Ai9* mice following 4-OHT treatment at P10.

A-D’ Immunostaining for TdTomato in Organ of Corti wholemounts of *Cdh5-CreERT2; Ctnnb1$^{flex/+}$; Ai9* mice at 1 month after 4-OHT treatment at P10. Following 4-OHT treatment, Cre activity in endothelial cells recombined the Ai9 Cre reporter allele to produce the fluorescent protein TdTomato (TdTom, red) in vessels of the of the modiolus (A-B’). TdTom localization indicating Cre activity was not observed in outer or inner hair cells counter stained with an anti-Myo7a antibody (green, A-A’, B-B’, C-C’). C-C’ show boxed region from B-B’ at high magnification). Rarely, evidence of Cre activity was observed in vascular endothelial cells in the absence of 4-OHT induction (D’, arrows). A-D show tile scans of stacked images through the thickness of the organ of Corti acquired on a spinning disk confocal microscope (Yokogawa, CSU22).

4-OHT treated *Cdh5-CreERT2; Ctnnb1$^{flex/+}$; Ai9* n= 4, untreated *Cdh5-CreERT2; Ctnnb1$^{flex/+}$; Ai9* n = 3. Scale bars: 50 μm.

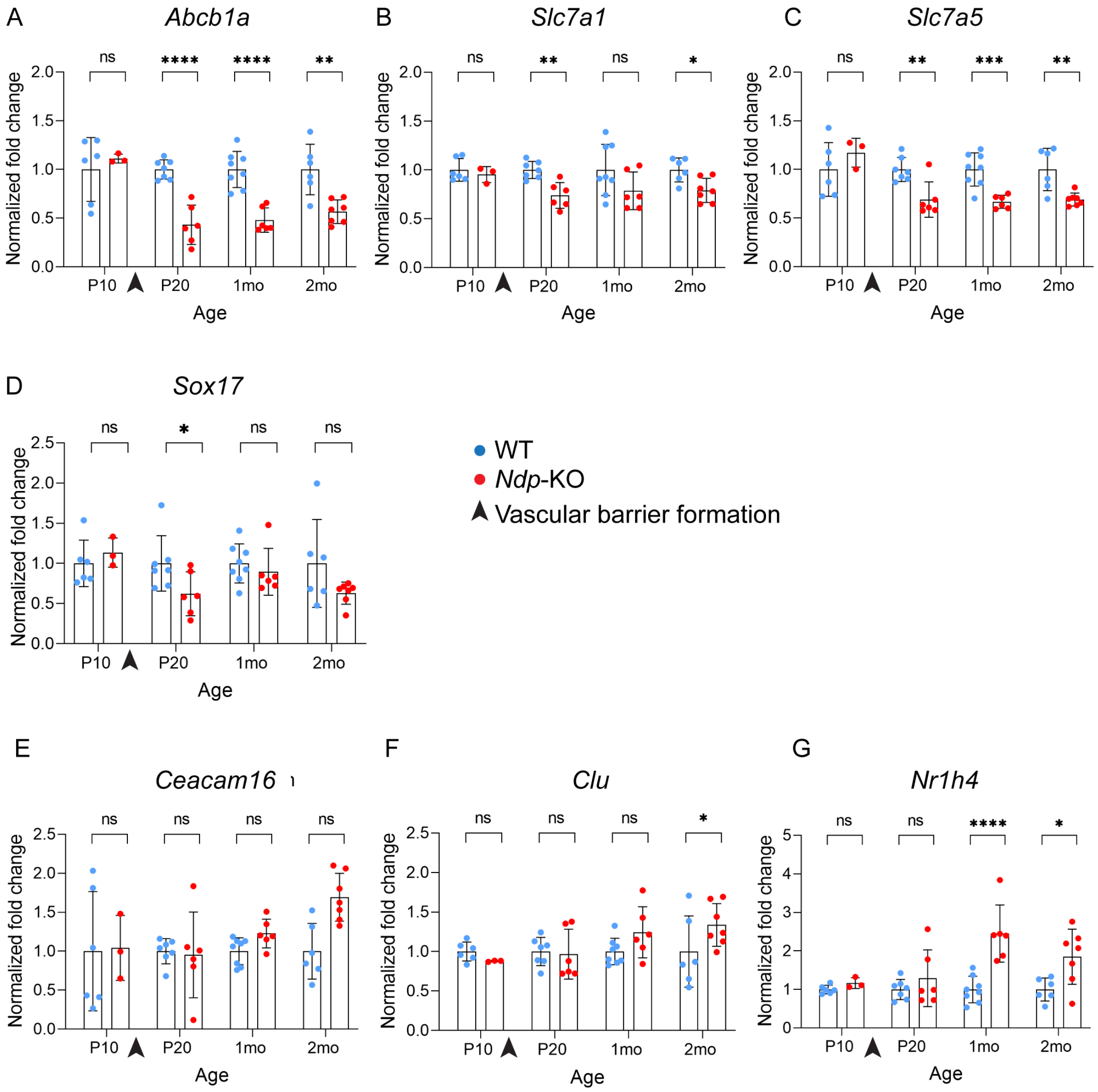
A
Abcb1a
B
Slc7a1
C
Slc7a5
D
Sox17
E
Ceacam16
F
Clu
G
Nr1h4
Normalized fold change
ns
****
**
*
***
P10
P20
1mo
2mo
Age
WT
Ndp-KO
Vascular barrier formation

Supplementary Figure 6: qRT-PCR analysis shows expression Norrie disease biomarkers in the mouse cochlea.

A-G: Timeline of expression of disease biomarker genes (previously identified in[1], [8]) in the WT and *Ndp*-KO mouse cochlea by qRT-PCR. Selected timepoints extend before and after vascular barrier formation (P12, arrowhead) Downregulation of vascular genes is apparent immediately after barrier formation in *Ndp*-KO mice.

qRT-PCR: P10 WT n = 6, *Ndp*-KO n = 3; P20 WT n = 7, *Ndp*-KO n = 6; 1 month WT n = 8, *Ndp*-KO = 6; 2 months WT n = 6, *Ndp*-KO n = 7. Statistical analysis by mixed-effects model and Sidak's post-hoc test. *p<0.05, **p<0.01, ***p<0.001.

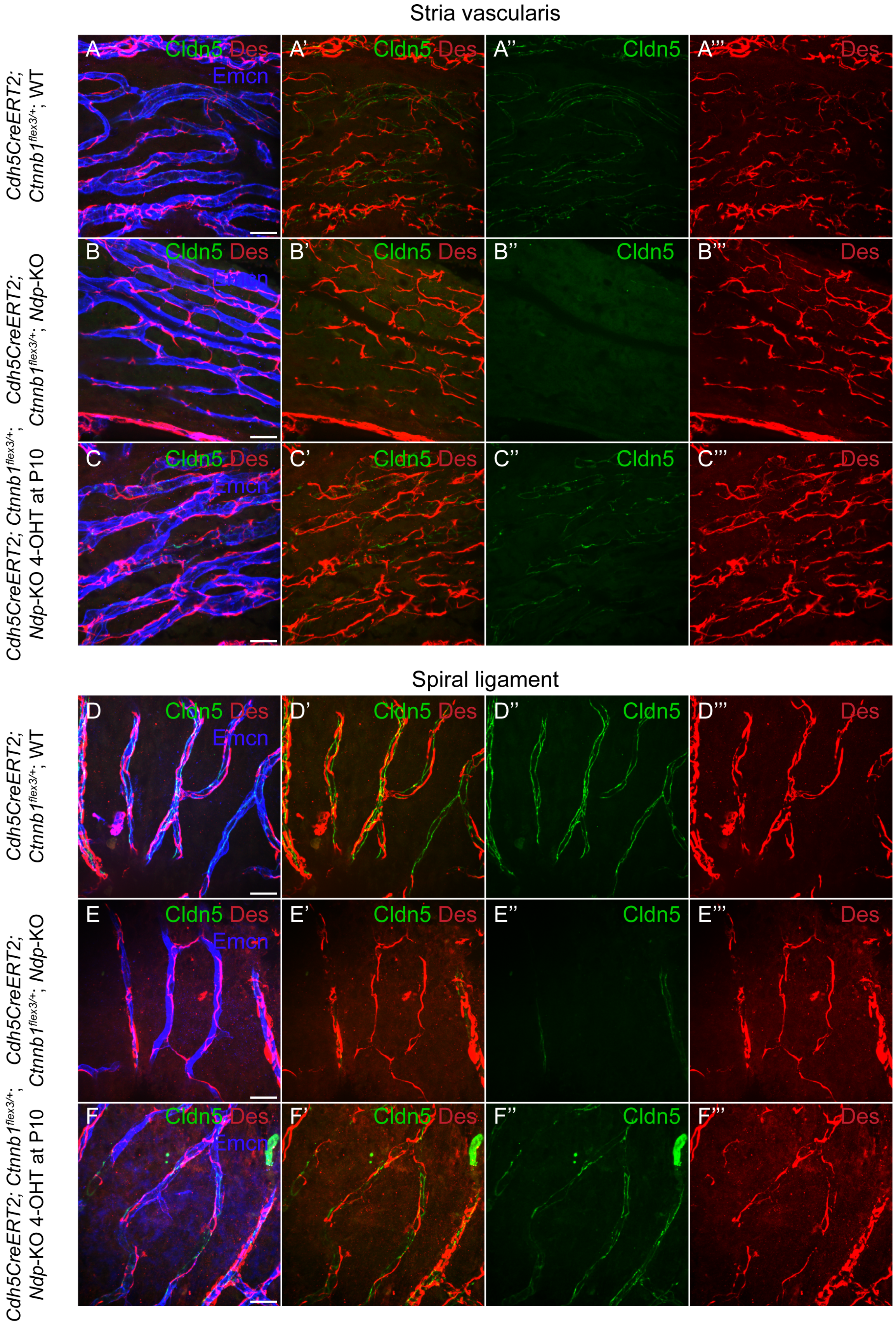
Stria vascularis
A Cldn5 Des Emcn
A’ Cldn5 Des
A’’ Cldn5
A’’’ Des
B Cldn5 Des Emcn
B’ Cldn5 Des
B’’ Cldn5
B’’’ Des
C Cldn5 Des Emcn
C’ Cldn5 Des
C’’ Cldn5
C’’’ Des
Spiral ligament
D Cldn5 Des Emcn
D’ Cldn5 Des
D’’ Cldn5
D’’’ Des
E Cldn5 Des Emcn
E’ Cldn5 Des
E’’ Cldn5
E’’’ Des
F Cldn5 Des Emcn
F’ Cldn5 Des
F’’ Cldn5
F’’’ Des
Cdh5CreERT2; Ctnnb1flex3/+; WT
Cdh5CreERT2; Ctnnb1flex3/+; Ndp-KO
Cdh5CreERT2; Ctnnb1flex3/+; Ndp-KO 4-OHT at P10

Supplementary figure 7: Analysis of lateral walls shows β-catenin stabilization in the vascular endothelial cells restored Cldn5 expression in the tight junctions between the endothelial cells and pericyte coverage in both the stria vascularis and spiral ligament.

A-F: Immunostaining for vascular tight junction protein Cldn5 and pericyte protein Desmin in stria vascularis (A-C) and spiral ligament (D-F) capillaries of 4-OHT treated *Cdh5CreERT2;Ctnnb1$^{flex3/+}$;Ndp*-KO and untreated control mice at 2 months.

A-F'' show tile scans of stacked images through the thickness of the stria vascularis (A-C) or spiral ligament (D-F'') acquired on a spinning disk confocal microscope (Yokogawa, CSU22).

Scale bars: 20 µm (A-F).

Cldn5 localization 2 months *Cdh5CreERT2;Ctnnb1$^{flex3/+}$;*WT n = 3, *Cdh5CreERT2;Ctnnb1$^{flex3/+}$;Ndp*-KO n = 5, 4-OHT treated *Cdh5CreERT2;Ctnnb1$^{flex3/+}$;Ndp*-KO n = 4; Des localization 2 months *Cdh5CreERT2;Ctnnb1$^{flex3/+}$;*WT n = 6, *Cdh5CreERT2;Ctnnb1$^{flex3/+}$;Ndp*-KO n = 5; 4-OHT treated *Cdh5CreERT2;Ctnnb1$^{flex3/+}$;Ndp*-KO n = 4.

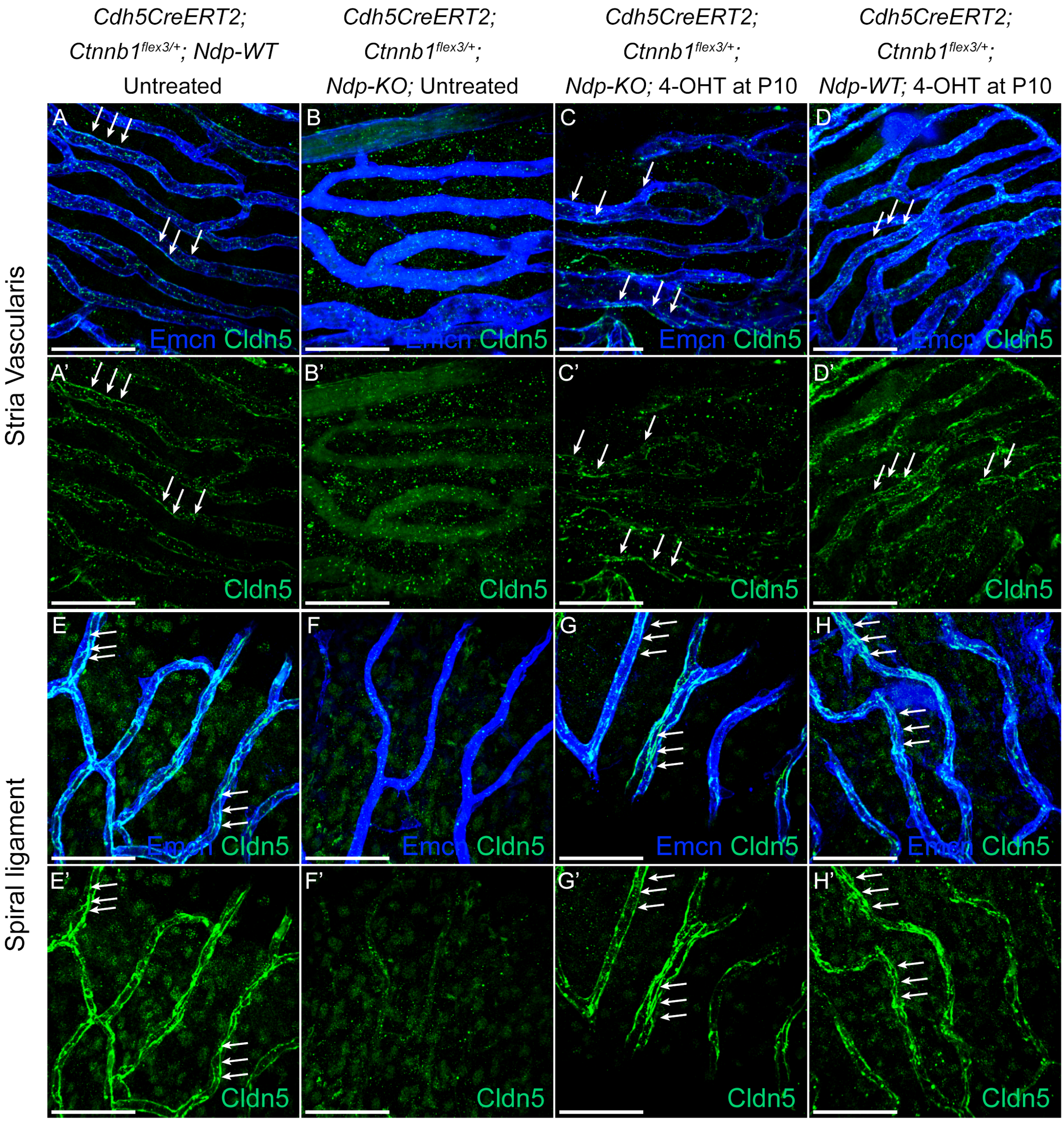
Cdh5CreERT2; Ctnnb1flex3/+; Ndp-WT Untreated
Cdh5CreERT2; Ctnnb1flex3/+; Ndp-KO; Untreated
Cdh5CreERT2; Ctnnb1flex3/+; Ndp-KO; 4-OHT at P10
Cdh5CreERT2; Ctnnb1flex3/+; Ndp-WT; 4-OHT at P10
Stria Vascularis
Spiral ligament
A
B
C
D
A'
B'
C'
D'
E
F
G
H
E'
F'
G'
H'
Emcn Cldn5
Cldn5

Supplementary Figure 8: β-catenin stabilization in the vascular endothelial cells restored Cldn5 localisation in the tight junctions between the endothelial cells in the stria vascularis and lateral wall.

A-H': Immunostaining for vascular tight junction protein Cldn5 in lateral wall whole mounts of 4-OHT treated *Cdh5CreERT2;Ctnnb1$^{flex3/+}$;Ndp*-KO and *Cdh5CreERT2;Ctnnb1$^{flex3/+}$;*WT and untreated control mice at 2 months showing stria vascularis (A-D') and spiral ligament (D-H') capillaries.
Cldn5 localization was observed at vascular tight junctions (arrows) in untreated *Cdh5CreERT2;Ctnnb1$^{flex3/+}$;*WT (A-A', E-E') and 4-OHT treated *Cdh5CreERT2;Ctnnb1$^{flex3/+}$;Ndp*-KO (C-C', G-G') and *Cdh5CreERT2;Ctnnb1$^{flex3/+}$;*WT (D-D', H-H') mice. It was not observed in untreated *Cdh5CreERT2;Ctnnb1$^{flex3/+}$;Ndp*-KO mice (B-B', F-F').
Vascular morphology and Cldn5 localization appeared similar in treated and untreated *Cdh5CreERT2;Ctnnb1$^{flex3/+}$;*WT mice with no obvious abnormalities.

A-H' show tile scans of stacked images through the thickness of the stria vascularis (A-D') or spiral ligament (E-H'') acquired on a spinning disk confocal microscope (Yokogawa, CSU22).

Cldn5 localization 2 months *Cdh5CreERT2;Ctnnb1$^{flex3/+}$;*WT n = 3, *Cdh5CreERT2;Ctnnb1$^{flex3/+}$;Ndp*-KO n = 5, 4-OHT treated *Cdh5CreERT2;Ctnnb1$^{flex3/+}$;Ndp*-KO n = 4, 4-OHT treated *Cdh5CreERT2;Ctnnb1$^{flex3/+}$;*WT n = 2.

Scale bars: 50 μm.

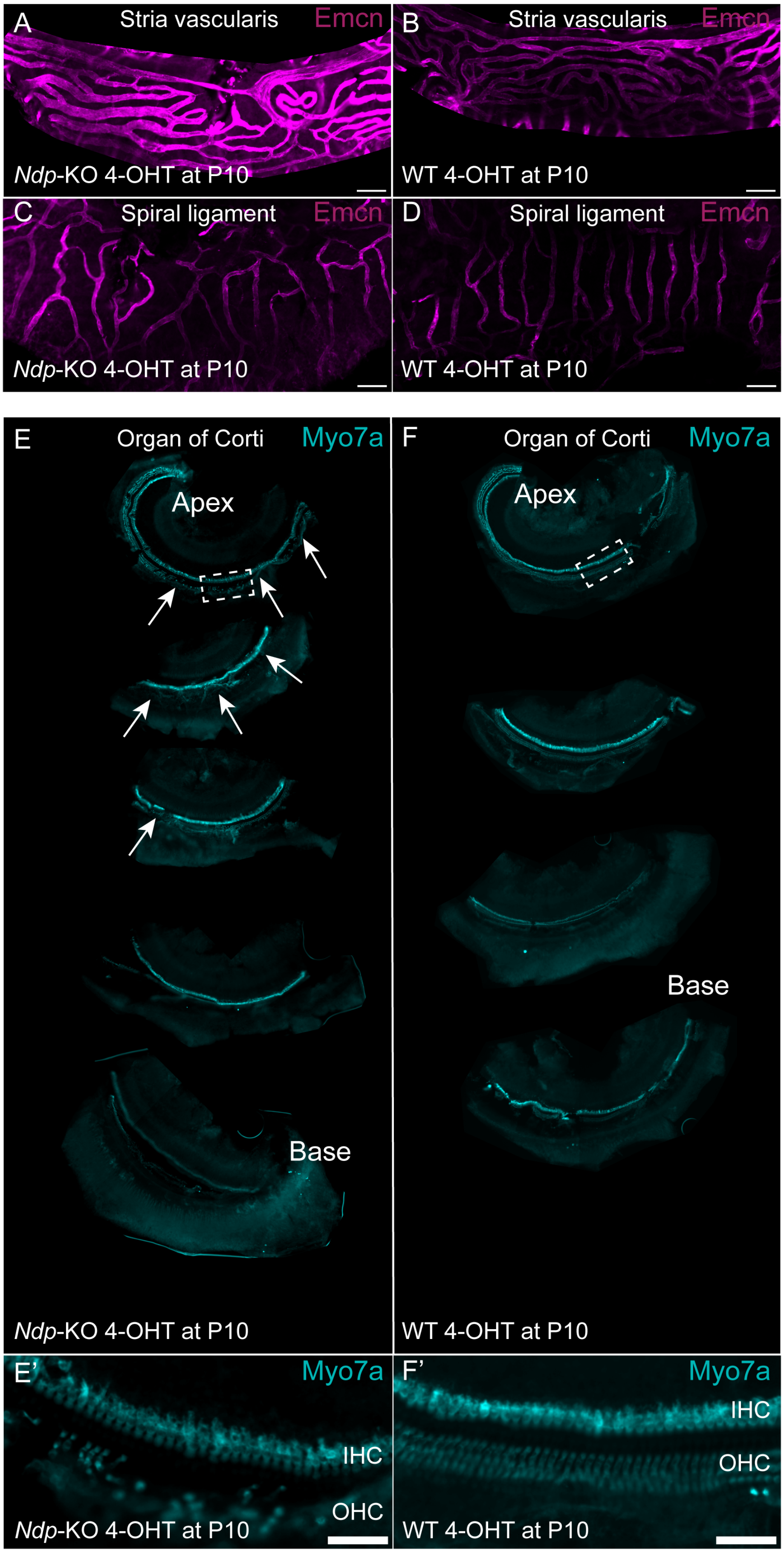
A
Stria vascularis
Emcn
Ndp-KO 4-OHT at P10
B
Stria vascularis
Emcn
WT 4-OHT at P10
C
Spiral ligament
Emcn
Ndp-KO 4-OHT at P10
D
Spiral ligament
Emcn
WT 4-OHT at P10
E
Organ of Corti
Myo7a
Apex
Base
Ndp-KO 4-OHT at P10
F
Organ of Corti
Myo7a
Apex
Base
WT 4-OHT at P10
E'
Myo7a
IHC
OHC
Ndp-KO 4-OHT at P10
F'
Myo7a
IHC
OHC
WT 4-OHT at P10

Supplementary figure 9: 4-OHT treatment in the absence of the *Cdh5CreERT2* and *Ctnnb1^{flex3}* alleles did not prevent cochlear vascular abnormalities and hair cell death.

A-D: Wholemounts of the lateral wall showing stria vascularis (A, B) and spiral ligament (C, D) capillary morphology by anti-Endomucin (Emcn, magenta) immunostaining in *Ndp*-KO and WT mice (lacking Cdh5CreERT2 and Ctnnb1^{flex3} alleles) at 2 months following 4-OHT treatment at P10. The *Ndp*-KO showed abnormally enlarged strial capillaries with elongated segments (A), compared to WT (B) which were not prevented by 4-OHT treatment alone. . A-D show tile scans of single plane images acquired on a spinning disk confocal microscope (Nikon Eclipse Ti2, Crest Optics).

E-F': Wholemounts of the organ of Corti with immunostaining for Myo7a showing inner hair cells (IHC) and outer hair cells (OHC) in Ndp-KO and WT mice (lacking Cdh5CreERT2 and Ctnnb1^{flex3} alleles) at 2 months following 4-OHT treatment at P10. Ndp-KO mice (E) showed OHC loss in the mid-apical region. E' and F' show boxed regions from C and D respectively.

4-OHT treated *Ndp*-KO n=4, 4-OHT treated WT n=1. Scale bars 50 µm.

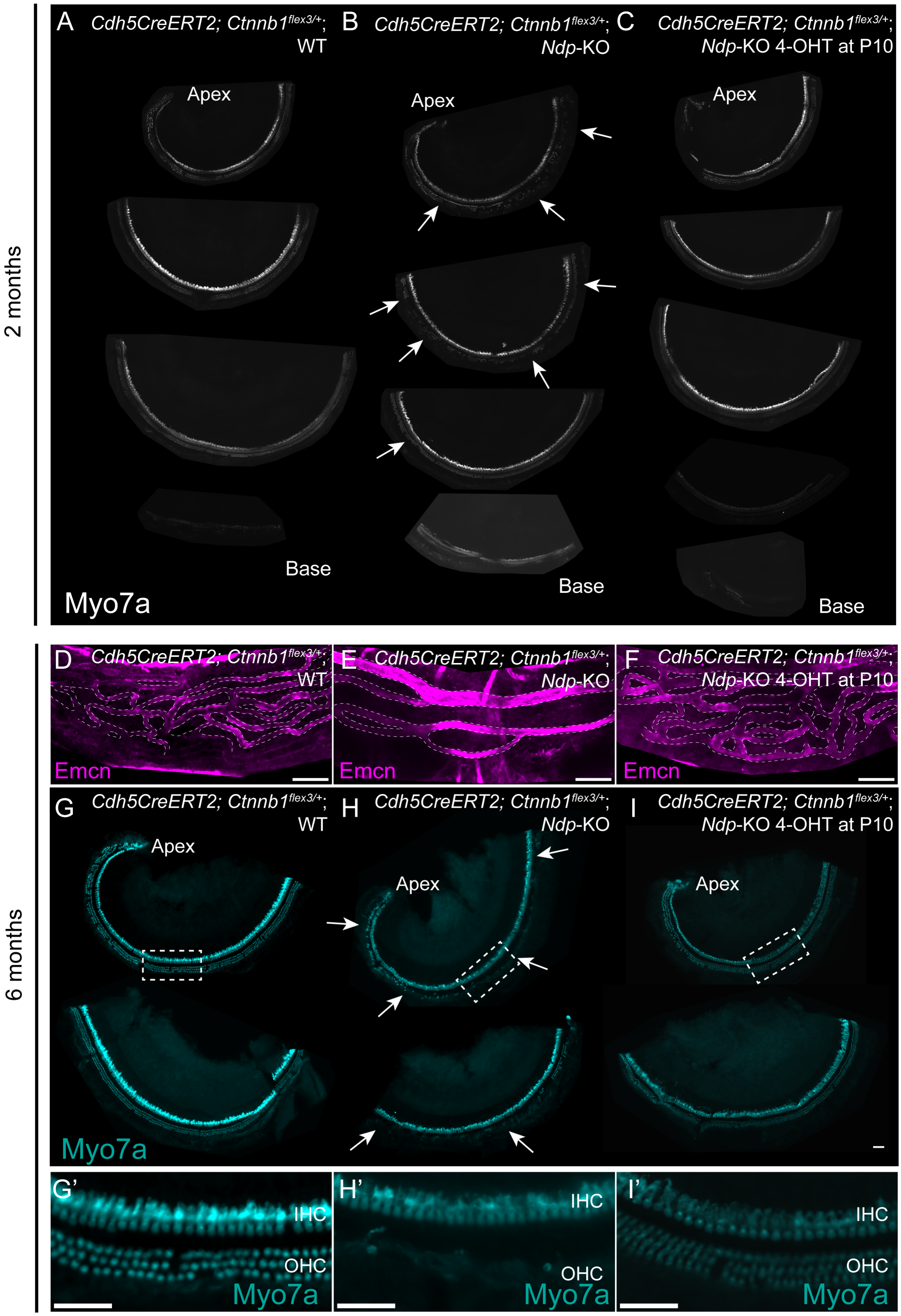
2 months
A Cdh5CreERT2; Ctnnb1flex3/+; WT
B Cdh5CreERT2; Ctnnb1flex3/+; Ndp-KO
C Cdh5CreERT2; Ctnnb1flex3/+; Ndp-KO 4-OHT at P10
Apex
Base
Myo7a
6 months
D Cdh5CreERT2; Ctnnb1flex3/+; WT
E Cdh5CreERT2; Ctnnb1flex3/+; Ndp-KO
F Cdh5CreERT2; Ctnnb1flex3/+; Ndp-KO 4-OHT at P10
Emcn
G Cdh5CreERT2; Ctnnb1flex3/+; WT
H Cdh5CreERT2; Ctnnb1flex3/+; Ndp-KO
I Cdh5CreERT2; Ctnnb1flex3/+; Ndp-KO 4-OHT at P10
G' H' I'
IHC
OHC

Supplementary figure 10: OHC death was prevented by vascular β-catenin stabilization.

A-C: Full-length organ of Corti wholemounts at 2 months, immunostained with anti-Myo7a antibody, labelling inner and outer hair cells. Images are stitched tile scans of single plane images taken on a fluorescent microscope (Zeiss Observer).

D-F: Lateral wall whole mounts showing stria vascularis capillary morphology by anti-Endomucin immunostaining at 6 months in treated and control mice. Images are stitched tile scans of single plane images acquired on a spinning disk confocal microscope (Yokogawa, CSU22).

G-I: Immunostaining for Myo7a showing inner hair cells (IHC) and outer hair cells (OHC) in organ of Corti wholemounts at 6 months. Images are stitched tile scans of single plane images taken on a fluorescent microscope (Zeiss Observer).

G'-I': Boxed region in G-I, showing IHC and OHC in organ of Corti wholemounts at 6 months.

(A-C) At 2 months: *Cdh5CreERT2;Ctnnb1*$^{flex3/+}$*;*WT n = 6, *Cdh5CreERT2;Ctnnb1*$^{flex3/+}$*;Ndp*-KO n = 6, 4-OHT treated *Cdh5CreERT2;Ctnnb1*$^{flex3/+}$*;*Ndp-KO n = 6.

(D-I) At 6 months *Cdh5CreERT2;Ctnnb1*$^{flex3/+}$*;*WT n = 3, *Cdh5CreERT2;Ctnnb1*$^{flex3/+}$*;Ndp*-KO n = 3, 4-OHT treated *Cdh5CreERT2;Ctnnb1*$^{flex3/+}$*;Ndp*-KO n = 2.

Scale bars: 50 μm (D-I).

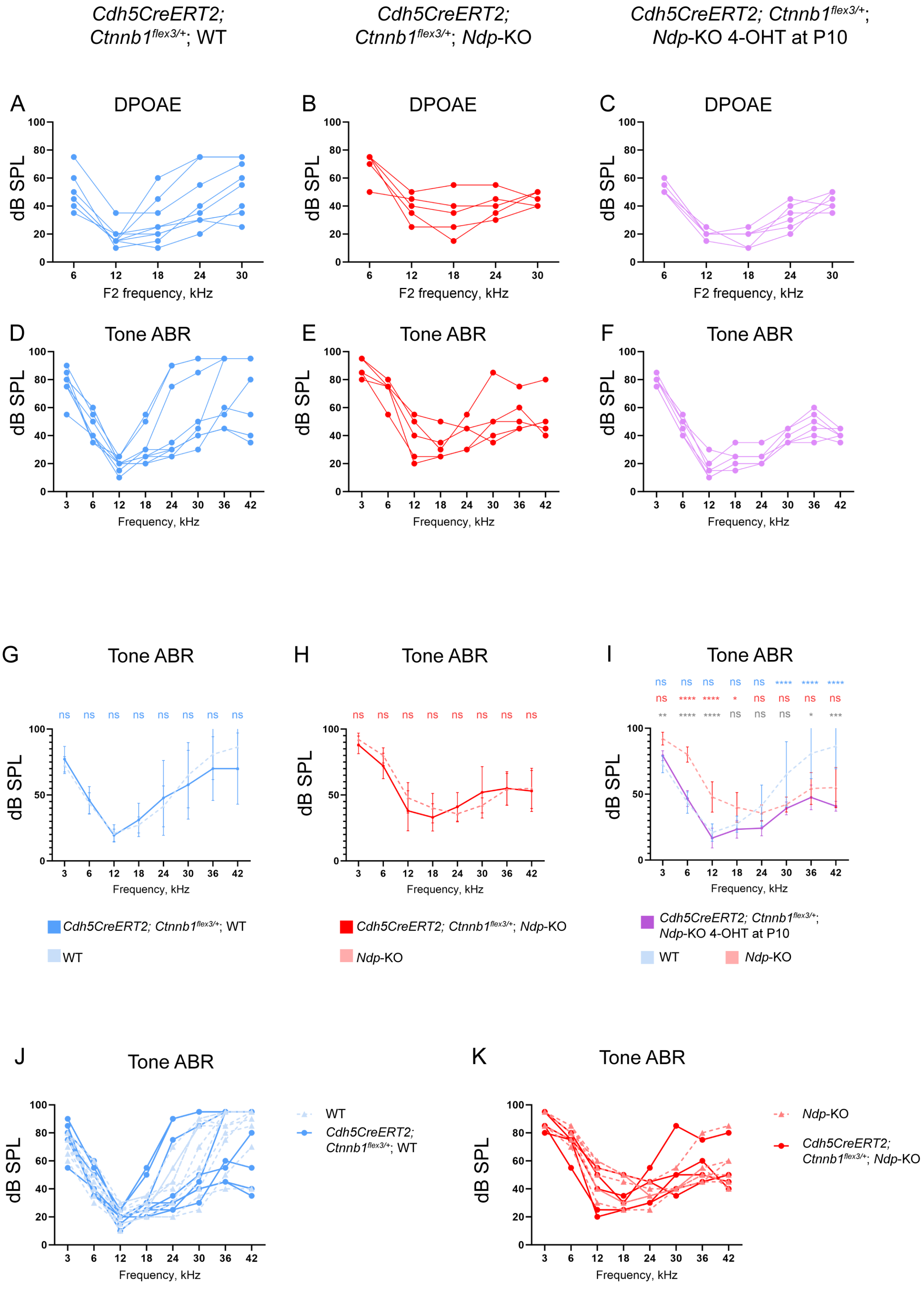

Cdh5CreERT2; Ctnnb1flex3/+; WT
Cdh5CreERT2; Ctnnb1flex3/+; Ndp-KO
Cdh5CreERT2; Ctnnb1flex3/+; Ndp-KO 4-OHT at P10
A
B
C
DPOAE
dB SPL
F2 frequency, kHz
D
E
F
Tone ABR
Frequency, kHz
G
H
I
ns
**
****
***
*
Cdh5CreERT2; Ctnnb1flex3/+; WT
WT
Cdh5CreERT2; Ctnnb1flex3/+; Ndp-KO
Ndp-KO
Cdh5CreERT2; Ctnnb1flex3/+; Ndp-KO 4-OHT at P10
J
K

Supplementary figure 11: Tests of auditory function at 3 months.

A-C: DPOAE threshold measurements of untreated *Cdh5CreERT2; Ctnnb1$^{flex3/+}$;* WT and *Cdh5CreERT2; Ctnnb1$^{flex3/+}$; Ndp*-KO mice and 4-OHT treated *Cdh5CreERT2; Ctnnb1$^{flex3/+}$; Ndp*-KO mice at 3 months, individual traces.

D-F: Pure tone ABR threshold measurements of untreated *Cdh5CreERT2; Ctnnb1$^{flex3/+}$;* WT and *Cdh5CreERT2; Ctnnb1$^{flex3/+}$; Ndp*-KO mice and 4-OHT treated *Cdh5CreERT2; Ctnnb1$^{flex3/+}$; Ndp*-KO mice at 3 months, individual traces.

G-H: Pure tone ABR thresholds of untreated *Cdh5CreERT2; Ctnnb1$^{flex3/+}$;* WT and *Cdh5CreERT2; Ctnnb1$^{flex3/+}$; Ndp*-KO compared to previously reported WT and *Ndp*-KO mice (dashed lines) (1). No significant difference was observed.

I: Pure tone ABR thresholds of 4-OHT treated *Cdh5CreERT2;Ctnnb1$^{flex3/+}$;Ndp*-KO mice compared to previously reported *Ndp*-KO and WT mice (dashed lines) (1). *Ndp*-KO mice had significantly elevated thresholds in the low frequency region. No significant difference was observed between the thresholds of WT and 4-OHT treated *Cdh5CreERT2;Ctnnb1$^{flex3/+}$;Ndp*-KO mice in this region.

J, K: Pure tone ABR threshold measurements of untreated *Cdh5CreERT2; Ctnnb1$^{flex3/+}$;* WT and *Cdh5CreERT2; Ctnnb1$^{flex3/+}$; Ndp*-KO mice as individual traces overlaid on those of previously reported untreated *Cdh5CreERT2; Ctnnb1$^{flex3/+}$;* WT and *Cdh5CreERT2; Ctnnb1$^{flex3/+}$; Ndp*-KO (dashed lines). The variability in ABR thresholds of individual mice in the high frequency region may be due to the presence of the age related hearing loss *Cdh23*^{ahl} allele in the BL6 background of the colony. The *Cdh23^{ahl}* allele is a hypomorphic silent single base change at the end of exon 7 that leads to preferential skipping of exon 7 in the transcript (2) the impact of this type of variant may be more variable than coding changes.

Data are shown as individual traces (A-F, J, K) or as mean ± SD (G – I). G, H analysed with Two-way ANOVA and Holm-Šídák's multiple comparisons test; I analysed with Two-way ANOVA and Tukey's multiple comparisons test.

*Cdh5CreERT2;Ctnnb1$^{flex3/+}$;*WT n = 9, *Cdh5CreERT2;Ctnnb1$^{flex3/+}$;Ndp*-KO n = 5, 4-OHT treated *Cdh5CreERT2;Ctnnb1$^{flex3/+}$;Ndp*-KO n = 6

WT n = 11, *Ndp*-KO n = 7 shown in light blue and light red (dashed lines) are ABR threshold data in G- K reported in our previous publication (1) (Source Data from Figure 8) and have been shown here for comparison. The mice in that study were derived from the same *Ndp^{tm1Wbrg}* (*Ndp^{-}*) colony as the mice in the current study and were assessed using the same auditory testing apparatus and methods.

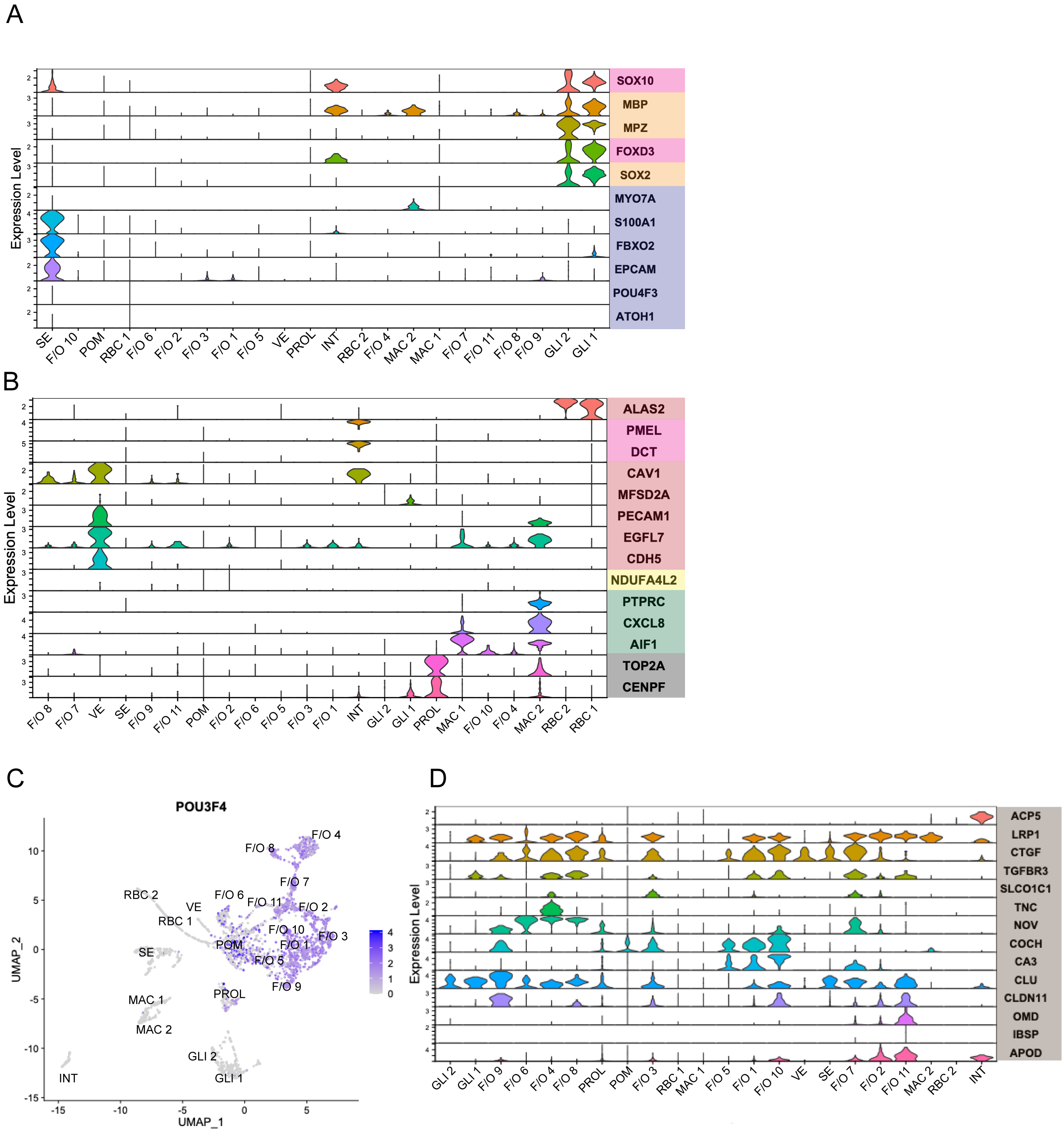
A
Expression Level
SOX10
MBP
MPZ
FOXD3
SOX2
MYO7A
S100A1
FBXO2
EPCAM
POU4F3
ATOH1
SE F/O 10 POM RBC 1 F/O 6 F/O 2 F/O 3 F/O 1 F/O 5 VE PROL INT RBC 2 F/O 4 MAC 2 MAC 1 F/O 7 F/O 11 F/O 8 F/O 9 GLI 2 GLI 1
B
Expression Level
ALAS2
PMEL
DCT
CAV1
MFSD2A
PECAM1
EGFL7
CDH5
NDUFA4L2
PTPRC
CXCL8
AIF1
TOP2A
CENPF
F/O 8 F/O 7 VE SE F/O 9 F/O 11 POM F/O 2 F/O 6 F/O 5 F/O 3 F/O 1 INT GLI 2 GLI 1 PROL MAC 1 F/O 10 F/O 4 MAC 2 RBC 2 RBC 1
C
POU3F4
UMAP_2
UMAP_1
F/O 4
F/O 8
F/O 7
RBC 2
F/O 6
F/O 11
F/O 2
VE
RBC 1
F/O 10
F/O 3
SE
POM
F/O 1
F/O 5
F/O 9
PROL
MAC 1
MAC 2
GLI 2
GLI 1
INT
D
Expression Level
ACP5
LRP1
CTGF
TGFBR3
SLCO1C1
TNC
NOV
COCH
CA3
CLU
CLDN11
OMD
IBSP
APOD
GLI 2 GLI 1 F/O 9 F/O 6 F/O 4 F/O 8 PROL POM F/O 3 RBC 1 MAC 1 F/O 5 F/O 1 F/O 10 VE SE F/O 7 F/O 2 F/O 11 MAC 2 RBC 2 INT

Supplementary figure 12: Identification of cell types in the 15 pcw human cochlea by single cell transcriptomic analysis.

A-B: Violin plots showing the expression of genes strongly expressed in glial (**GLI1, GLI2:** *SOX10, FOXD3, MBP, MPZ* (3, 4)) and sensory epithelial cell (**SE***: EPCAM, S100A1, FBXO1* (5-7)) cells (A) and genes expressed in red blood cells (**RBC1, RBC2:** *ALAS2* (8)), intermediate cell / melanocyte (**INT***: SOX10, FOXD3, PMEL, DCT, CAV1*), vascular endothelial cell (**VE***: CDH5, PECAM1 and EGFL7* (9-11), pericyte (**PER:** *NDUFA4L2* (12)), macrophage (**MAC1, MAC2:** *AIF1, PTPRC(CD45)* (13, 14)) and proliferating cell (**PROL:** *CENPF* and *TOP2A* (15-17)) (B) clusters in the 15 pcw human cochlea.

C: UMAP plot showing the expression of *POU3F4* (a marker of periotic mesenchyme (18)) in the 15pcw cochlea.

D: Violin plots showing the expression of selected fibrocyte and osteoblast (**F/O:** *LRP1*, *CTGF*, *COCH*, *CLU*, *TGFBR3, SLCO1C1*, *TNC*, *NOV,OMD* and *CA3* (17, 19)) markers in the 15 pcw human cochlea.

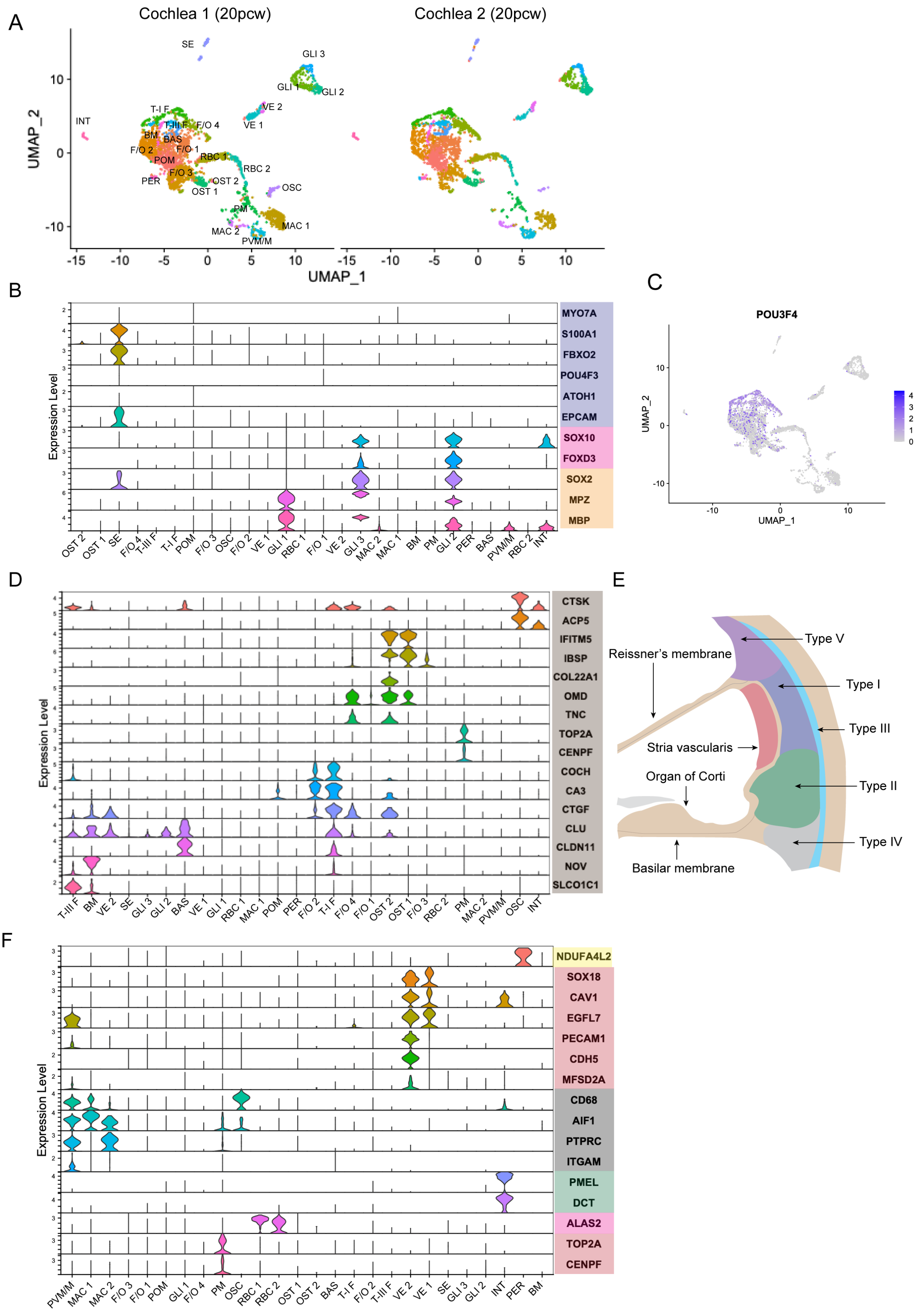
A
Cochlea 1 (20pcw)
Cochlea 2 (20pcw)
UMAP_2
UMAP_1
SE
GLI 3
GLI 1
GLI 2
VE 2
VE 1
INT
T-I F
T-III F
F/O 4
BM
BAS
F/O 2
F/O 1
POM
RBC 1
RBC 2
F/O 3
PER
OST 2
OST 1
OSC
PM
MAC 2
MAC 1
PVM/M
B
Expression Level
MYO7A
S100A1
FBXO2
POU4F3
ATOH1
EPCAM
SOX10
FOXD3
SOX2
MPZ
MBP
C
POU3F4
D
CTSK
ACP5
IFITM5
IBSP
COL22A1
OMD
TNC
TOP2A
CENPF
COCH
CA3
CTGF
CLU
CLDN11
NOV
SLCO1C1
E
Reissner's membrane
Type V
Type I
Type III
Stria vascularis
Organ of Corti
Type II
Basilar membrane
Type IV
F
NDUFA4L2
SOX18
CAV1
EGFL7
PECAM1
CDH5
MFSD2A
CD68
AIF1
PTPRC
ITGAM
PMEL
DCT
ALAS2
TOP2A
CENPF

Supplementary Figure 13: Identification of cell types within the 20pcw human cochlea by single cell transcriptomic analysis.

A: UMAP plots of individual 20pcw human cochlea samples. Both 20pcw cochlea samples showed a similar distribution of cell types.

B: Violin plots showing expression of gene strongly expressed in the sensory epithelium (**SE:** *EPCAM, S100A1, FBXO1* (5-7) and glial cells (**GLI 1-3:** MBP, MPZ (3, 4)) and sensory hair cell (ATOH1, MYO7A or POU4F3), supporting cell (SOX2) markers and in the 20pcw human cochlea.

C: UMAP plot showing the expression of *POU3F4* (a marker of cells originating from periotic mesenchyme (18))) in the 20pcw cochlea

D: Violin plots showing expression of selected fibrocyte and osteoblast (**OST 1, 2:** *OMD, IBSP, IFITM5*) (20-22) markers in the 20pcw human cochlea. Fibrocyte subtypes include Type I fibrocytes (**T- I F**: low *CLDN11, CTGF*, *COCH*+ve, *CA3*), Type III fibrocytes of the spiral ligament (**T- III F**;:*SLCO1C1*+ve), precursors of basal cells (**BAS**) (high *CLDN11*, *CLU*) and cells of the basilar membrane (**BM:** *NOV*+ve) (15, 16, 19, 23, 24) and 4 unknown fibrocyte clusters (**F/O 1- 4**) of which one expressed *TNC* encoding a protein of the basilar membrane and spiral lamina (**F/O4**) (25).

E: Schematic showing the location of fibrocyte subtypes in a cross section of the mammalian cochlea lateral wall.

F: Violin plots showing genes strongly expressed in red blood cells (RBC1,2: *ALAS2* (8)), pericytes (**PER:** *NDUFA4L2* (12)), vascular endothelial cells (**VE 1, 2:** *CDH5, PECAM1, SOX18*, *CAV1* and *EGFL7(9-11, 26, 27)*) macrophages (**MAC 1-2:** AIF1, PTPRC (13, 14)), intermediate cells / melanocytes (**INT:** *SOX10, FOXD3, PMEL, DCT, CAV1)* and proliferating cells (*TOP2A*, *CENPF* (15-17)) in the 20pcw human cochlea. One cluster of macrophages was identified as perivascular macrophage-like-melanocytes of the stria (**PVM/M:** *AIF1*, *PTPRCCD68, ITGAM*) (28, 29),another as osteoclasts (**OSC**: *CD68, AIF1, CTSK*, *ACP5;* see D) (30, 31) and a third as proliferating macrophages (PM: TOP2A, CENPF, CTSK, ACP5 (15-17))).

-

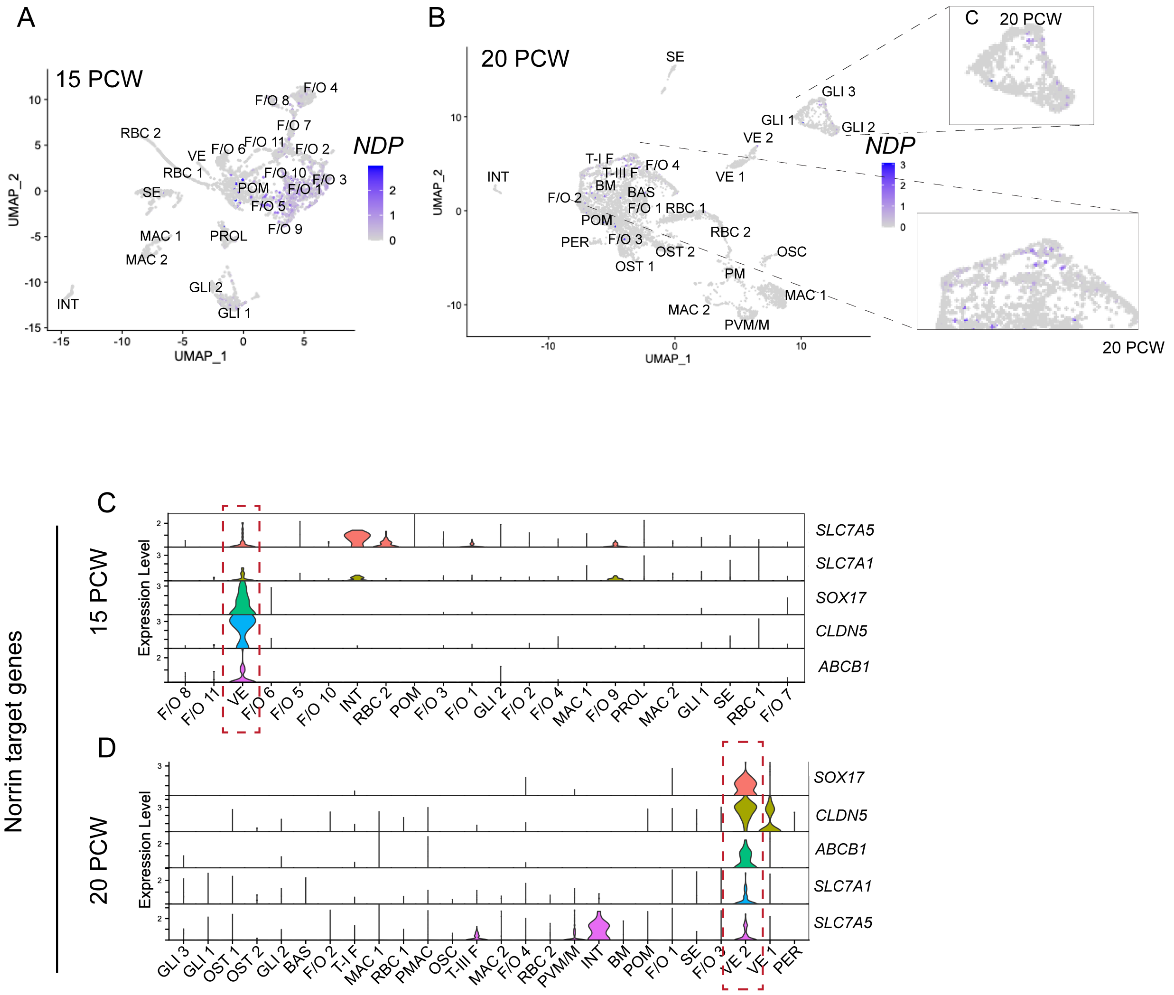

Supplementary Figure 14: Single cell transcriptomic analysis of the human cochlea shows expression of NDP and norrin target genes consistent with vasculature being a site of direct norrin signalling.

A: UMAP plot showing *NDP* expression in the human cochlea at 15 pcw. See Fig 6 B.

B: UMAP plot showing *NDP* expression in the human cochlea at 20 pcw. See Fig 6 C.

C-D: Violin plots showing expression of Norrin target genes in the 15 pcw (C) and 20pcw (D) human cochlea.

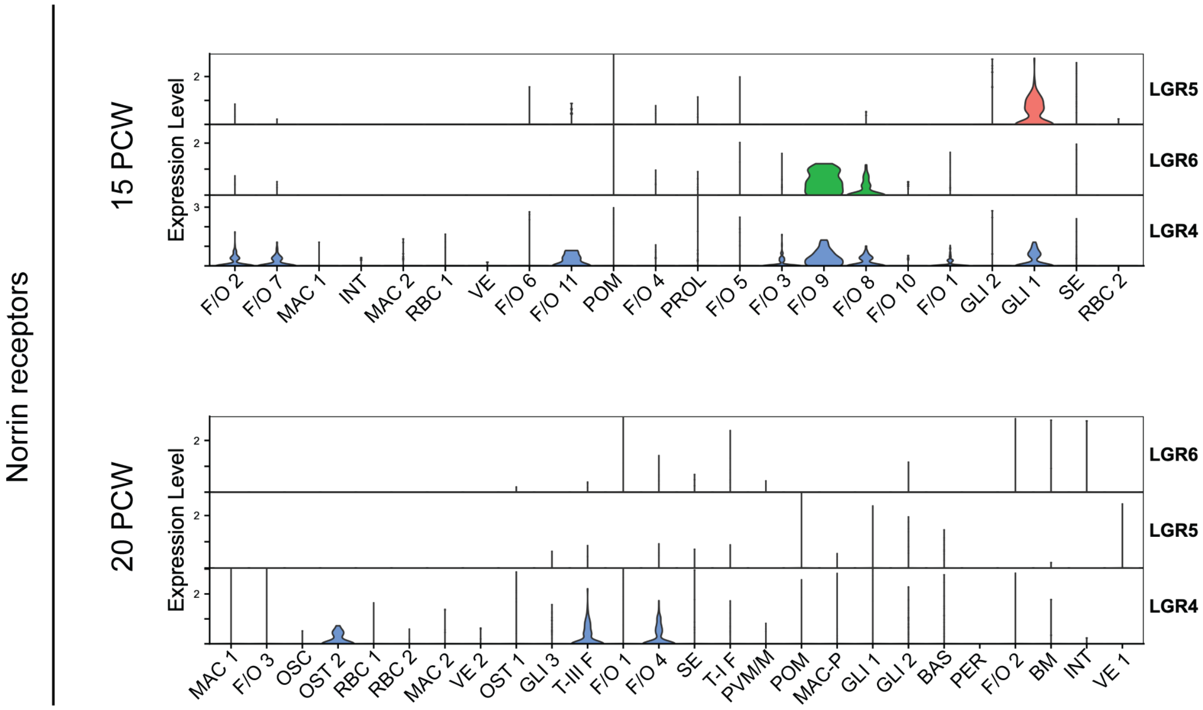

Supplementary Figure 15: Violin plots showing the expression of the norrin receptors LGR4-6 in 15pcw and 20pcw human cochleas by single cell transcriptomic analysis.

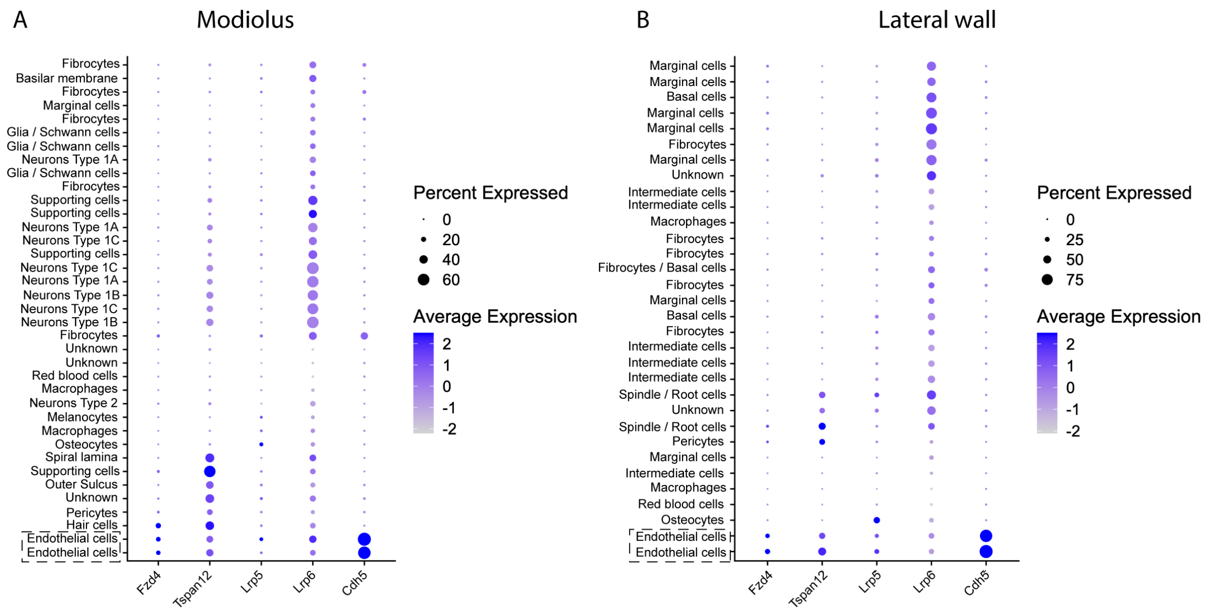

Supplementary Figure 16: Dot plots showing the expression of the norrin receptors *Fzd4, Tspan12, Lrp5* and Lrp6 in the adult mouse cochlea.
Dot plot prepared using scRNA seq data of the adult mouse cochlea modiolus and lateral wall from GEO database: accession numbers GSM5124291, GSM5124292, GSM5124293, GSM5124294, GSM5124299, GSM5124300, GSM5124301 and GSM5124302. Clustering and cluster annotation as described in (1)

Dataset S1: Cluster marker genes for the 15pcw human cochlea

Dataset S2: Cluster marker genes for 20pcw human cochleas

Supplementary methods

Animal experiments

Animal studies were carried out after University College London and King's College London Ethics Review and in accordance with UK Home Office regulations and the UK Animals (Scientific Procedures) Act of 1986 under UK Home Office license. Mice were kept at 12 hours light, 12 hours dark cycle and provided food and water *ad libitum.*

Mice carrying the *$Ndp^{tm1Wbrg}$* (*Ndp^{-}*) allele were provided by Prof W. Berger (32). Heterozygous *$Ndp^{+/-}$* females were crossed with wildtype (WT) C57BL/6J males from Charles River to maintain a largely C57BL/6 background. *$Ndp^{-/-}$* females are known to be infertile (33). *$Ndp^{y/-}$* males or *$Ndp^{-/-}$* females (referred to as *Ndp*-KO) were compared to age matched *$Ndp^{y/+}$* males or $Ndp^{-/-}$ females (referred to as *WT*).

Mouse lines carrying the *Cdh5CreERT2* transgene (34) and the *Ai9* Cre reporter allele (referred to as Ai9 in this manuscript) (35) on predominantly C57BL/6 backgrounds were provided by Prof D. Long (UCL). Mice carrying the *$Ctnnb1^{tm1Mmt}$* allele (36) (referred to as *$Ctnnb1^{flex3}$* in this manuscript) on a predominantly C57BL/6 background were provided by Prof J.P. Martinez-Barbera (UCL). We generated a *$Cdh5CreERT2^{tg/tg}$; $Ctnnb1^{flex3/flex3}$* homozygous line (Flex3 is an abbreviation of floxed exon 3) by breeding. Males from this line were crossed with fertile *$Ndp^{+/-}$* females to generate experimental F1 generation pups with a consistent genetic background. From these litters *$Cdh5CreERT2;Ctnnb1^{flex3/+};Ndp$-KO* or *$Cdh5CreERT2;Ctnnb1^{flex3/+};WT$* mice were used as control groups for analyses on the effect of β-catenin stabilization by tamoxifen induction on Norrie disease pathology.

Genomic DNA was isolated from ear or tail biopsies and genotypes determined by PCR (MyFi Mix BIO-25050), using primers in Table S1

**Primer**	**Forward**	**Reverse**	**Amplicon, bp**
gNdp	GTATTGCATCCATATTTCTTGG	CTCTCCATCCCCTGACAAGGA	**WT:** 550<br>***Ndp*-KO** 1500
Cre	ATGTCCAATTTACTGACCG	CGCCGCATAACCAGTGAAAC	~300
Ctnnb1	AGAATCACGGTGACCTGGGTTAAA	CATTCATAAAGGACTTGGGAGGTGT	**WT**: 600<br>**flex3:** ~700
Ai9; WT	AAGGGAGCTGCAGTGGAGTA	CCGAAAATCTGTGGGAAGTC	297
Ai9; Mutant	CTGTTCCTGTACGGCATGG	GGCATTAAAGCAGCGTATCC	~200

Table S1: Genotyping primers

Mouse pups were administered with a 2 µg/ul solution of 4-hydroxytamoxifen (Sigma, 4-OHT) in sterile sunflower oil by intraperitoneal injection at a final weight adjusted dose of 5 ug/g bodyweight. Mice were monitored regularly until 1 or 2 months of age. No adverse effects were observed.

Tissue processing

At 1 or 2 months of age mice were sacrificed by cervical dislocation and auditory bullae (inner ear separated from the temporal bone) were isolated. The apex of the cochlea was perforated and 4% paraformaldehyde (PFA) in PBS (w/v) injected through the round and oval windows. Fixation was continued by immersion of the cochlea in PFA for 90 minutes, followed by decalcification in 4% EDTA in PBS (w/v), pH 7.4 for 72 hours. Cochleas for cryosection were further processed by successive equilibration in 15% Sucrose (w/v in PBS) and 30% Sucrose (w/v), incubation in 1:1 mixture of OCT (Tissue-Tek) and 30% Sucrose for 4 hours on a rotating nutator followed by embedding in OCT and freezing on dry ice. Samples were stored at -80°C, and sectioned on a cryostat at 10 µm. Sections were collected on Superfrost-plus glass slides and stored at -80°C until staining. Eyes were isolated, the cornea was perforated and eyes were fixed by immersion in 4% PFA for 90 minutes. The eyes were enucleated and the retina isolated.

RNA extraction

Inner ears were isolated from the temporal bone, the vestibular region was broken off using watchmaker forceps and the cochleas snap frozen. Total RNA was extracted using a modification of a published protocol (37) (TRI Reagent 93289-25ML Sigma-Aldrich, DirectZol kit). RNA was eluted in 30µl of nuclease-free water and quantified on the NanoDropTM 2000 (Thermo Scientific).

qRT-PCR

cDNA was synthesised from 300-500 ng RNA using RevertAid H Minus First Strand cDNA Synthesis kit (K1631) with random hexamers according to manufacturer's instructions. cDNA equivalent to 1 ng of RNA was used per reaction for gene expression analysis with PowerSYBR Green PCR Master mix (436759) and relevant primers (Table S2).

Gene/ Primer ID	Forward	Reverse	Amplicon, bp
Abcb1a	GCGACTCCGATACATGGTT T	ACCCTGTAGCCCCTTTCACT	134
Actin-b	TGTTACCAACTGGGACGAC A	CTGGGTCATCTTTTCACGGT	139
Ceacam1 6	ATGAAAATGCCATTGACCT GGTA	TGTGTCCGTAGCCCACCT	376
Cldn5	TTAAGGCACGGGTAGCACT CACG	TTAGACATAGTTCTTCTTGTCG TAATCG	320
Clu	CCTTCCAGTCGAAGATGCT C	TGTGATGGGGTCAGAGTCAA	209
Nr1h4	AGGGAGAAAACGGAACTCA CGG	CCGCCGAACGAAGAAACATGG	283
Plvap	GTGGTTGGACTATCTGCCT C	ATAGCGGCGATGAAGCGA	188
Slc7a1	TTCGGTTATGGGATCTGGC ACAGT	TTTGCACTGGTCCAAGTTGCTG TC	87
Slc7a5	CTGGTCTTCGCCACCTACT T	GCCTTTACGCTGTAGCAGTTC	128
Sox17	GCACAGCAGAACCCAGATC T	CCGGTACTTGTAGTTGGGGT	156

Table S2: qRT-PCR primers

Immunohistochemistry

Whole mount preparations of the lateral wall and organ of Corti of decalcified cochlea were prepared and stained as previously described (38). The following antibodies were used: anti-endomucin (1:100, rat, Sc53941), anti-desmin (1:300, rabbit, 165201-AP) anti-Cldn5 Alexa Fluor 488 (1:100, mouse, 352588 Invitrogen), anti-Cldn5 (1:100, rabbit 34-1600 Invitrogen), anti-Myo7A (1:100, rabbit, 25-6790 Proteus Biosciences), anti-Pou4f3 (1:100, mouse, Sc81980), anti-TdTomato (1:100, goat, A121690), anti-β-catenin (1:1000, rabbit, ab16051 Abcam), anti-Estrogen Receptor (1:100, rabbit, ab16660) goat anti-mouse IgG Alexa Fluor 488 (A11001; Life Technologies), donkey anti-mouse IgG Alexa Fluor 594 (A21203; Life Technologies), goat anti-rat IgG Alexa Fluor 647 (A21247; ThermoFisher), donkey anti-rabbit IgG Alexa Fluor 488 (A21206; Life Technologies), donkey anti-rabbit IgG Alexa Fluor 555 (A31572; ThermoFisher), goat anti-rabbit IgG Alexa Fluor 568 (A11036; ThermoFisher). Lateral walls were mounted intact while organs of Corti were divided into 4-5 pieces. Cryosections for anti β-catenin immunostaining were treated with antigen retrieval (Tris-EDTA pH9 at 95°C for 2 minutes) prior to immunostaining.

Fluorescent tracer assay

To assess vascular barrier integrity, mice at 2 months of age were injected in the tail vein with 50µl of Fluorescein isothiocyanate-conjugated bovine serum albumin (FITC-BSA; A9771, Sigma-Aldrich) dissolved in PBS at 5% concentration. After 3 hours, mice were sacrificed,

cochleae isolated and the vasculature counterstained with an anti-endomucin antibody and histological analysis performed as described above.

Image analysis and Quantification of OHC death

Images were captured using a spinning disk confocal microscope (Yokogawa, CSU22) or fluorescence microscope (Zeiss Observer). Dissected organs of Corti stained with anti-Myo7a and Phalloidin were imaged as overlapping tiles and stitched into a single plane image. Hair cell survival was quantified as previously described (38). The length of each organ of Corti was measured using the Measure_line plugin, (https://masseyeandear.org/research/otolaryngology/eaton-peabody-laboratories/histology-core) and divided into 8 equal pieces from apex to base corresponding to tonotopic frequencies as follows: region 1/8 (3.1-6.1 kHz); region 2/8 (6.1-10.0 kHz), region 3/8 (10.0-15.0 kHz), region 4/8 (15.0-21.6 kHz), region 5/8 (21.6-30.2 kHz), region 6/8 (30.2-41.3 kHz), region 7/8 (41.3-55.9 kHz), region 8/8 (55.9-74.8 kHz). Within each piece, three non-overlapping 200 µm long rectangular regions were sampled. The numbers of surviving (Myo7a positive cells) and dead OHCs (phalloidin negative empty sockets of Deiters' cells and loss of OHC nucleus) were determined and the percent survival calculated. Integrity of the organ of Corti was confirmed by phalloidin staining of the whole sample.

Auditory electrophysiology

Auditory Brainstem Responses (ABR), Distortion Product Otoacoustic Emissions (DPOAE) and Endocochlear Potentials of treated and control mice were measured as described previously (38).

Collection and dissociation of Human cochlear samples

Human foetal cochlea samples were obtained from the Joint Medical Research Council UK (grant G0700089)/Wellcome Trust (grant GR082557) Human Developmental Biology Resource (http://www.hdbr.org/, HBDR) with ethics approval. Samples at 15 and 20 post-conception weeks (pcw) were analysed. Samples were anonymised by the HDBR prior to use in our study. Samples were processed on the day of collection. The temporal bone containing the cochlea was dissected in PBS. The otic capsule was broken open using forceps and the entire membranous labyrinth and modiolus were isolated. Each sample was transferred to a sterile tube containing 3 ml of enzyme mix (Miltenyi Neurosphere dissociation kit: 2880 µl Buffer X, 75 µl Enzyme P, 30 µl Buffer Y, 15 µl Enzyme A), incubated at 37°C for 60 minutes, rotating, followed by manual dissociation using a pipette and 1-2 further incubation steps at 37° until cells were completely dissociated. The cell suspension was passed through a 40µm cell strainer, diluted with an equal volume of 0.04% BSA-PBS. The cell suspension was

centrifuged at 200 rcf (200 x g), 4°C for 5 minutes to pellet the cells. The supernatant was removed, and cells resuspended in 0.04% BSA-PBS and maintained on ice. Cells were quantified and cell viability assessed using Trypan blue stain on a haemocytometer. Samples with a viability of 65% or higher were used for scRNA sequencing.

scRNA sequencing and analysis

Single Cell RNA libraries were generated using the Chromium Single Cell 3' Reagent Kit v3.1 (10X Genomics, CA, USA), with a target capture of ~10,000 cells per sample, following standard manufacturer's specifications. Libraries were evaluated for quality and sequenced on an Illumina platform. Raw sequence data was processed using the 10X Genomics' Cellranger software to generate final digital gene expression matrices. Data was analysed using the Seurat package. Cells expressing more than 200 and fewer than 8000 genes and with mitochondrial gene percentages less than 40% were retained for further analysis. The SoupX (39) tool was used to remove ambient RNA contamination. A total of 6077 cells from two 20 pcw cochlea samples and 2715 cells from one 15 pcw cochlea were analysed. Cells were clustered and top markers for all clusters identified using the FindAllMarkers command. Cell identity was assigned to each cluster based on markers described in the literature. Raw data are available on Array Express E-MTAB-14063

Statistics

The number of mice (n) used for each experiment and statistical tests used are stated in the figure legends. Error bars always represent standard deviation (SD). n indicates biological replicates throughout the study. Animals and tissue sample treatment groups were not blinded for analysis except for auditory electrophysiology where genotypes were masked. Some samples used for immunostaining analysis were omitted due to damage during dissection. Some samples were excluded prior to qRTPCR due to poor yield of total RNA or low purity.

1. V. Pauzuolyte *et al.*, Systemic gene therapy rescues retinal dysfunction and hearing loss in a model of Norrie disease. *EMBO Mol Med* 10.15252/emmm.202317393, e17393 (2023).
2. K. Noben-Trauth, Q. Y. Zheng, K. R. Johnson, Association of cadherin 23 with polygenic inheritance and genetic modification of sensorineural hearing loss. *Nat Genet* **35**, 21-23 (2003).
3. I. Breuskin *et al.*, Glial but not neuronal development in the cochleo-vestibular ganglion requires Sox10. *J Neurochem* **114**, 1827-1839 (2010).
4. E. J. Ryu *et al.*, Misexpression of Pou3f1 results in peripheral nerve hypomyelination and axonal loss. *J Neurosci* **27**, 11552-11559 (2007).

5. R. Hertzano *et al.*, Cell type-specific transcriptome analysis reveals a major role for Zeb1 and miR-200b in mouse inner ear morphogenesis. *PLoS Genet* **7**, e1002309 (2011).
6. A. G. Coppens, R. Kiss, C. W. Heizmann, B. W. Schafer, L. Poncelet, Immunolocalization of the calcium binding S100A1, S100A5 and S100A6 proteins in the dog cochlea during postnatal development. *Brain Res Dev Brain Res* **126**, 191-199 (2001).
7. B. H. Hartman, R. Durruthy-Durruthy, R. D. Laske, S. Losorelli, S. Heller, Identification and characterization of mouse otic sensory lineage genes. *Front Cell Neurosci* **9**, 79 (2015).
8. K. Meguro, K. Igarashi, M. Yamamoto, H. Fujita, S. Sassa, The role of the erythroid-specific delta-aminolevulinate synthase gene expression in erythroid heme synthesis. *Blood* **86**, 940-948 (1995).
9. D. Vestweber, VE-cadherin: the major endothelial adhesion molecule controlling cellular junctions and blood vessel formation. *Arterioscler Thromb Vasc Biol* **28**, 223-232 (2008).
10. L. H. Parker *et al.*, The endothelial-cell-derived secreted factor Egfl7 regulates vascular tube formation. *Nature* **428**, 754-758 (2004).
11. H. M. Delisser, H. S. Baldwin, S. M. Albelda, Platelet Endothelial Cell Adhesion Molecule 1 (PECAM-1/CD31): A Multifunctional Vascular Cell Adhesion Molecule. *Trends Cardiovasc Med* **7**, 203-210 (1997).
12. C. Mesa-Ciller *et al.*, Unique expression of the atypical mitochondrial subunit NDUFA4L2 in cerebral pericytes fine tunes HIF activity in response to hypoxia. *J Cereb Blood Flow Metab* **43**, 44-58 (2023).
13. D. De Leon-Oliva *et al.*, AIF1: Function and Connection with Inflammatory Diseases. *Biology (Basel)* **12** (2023).
14. Y. Dong *et al.*, Differential fates of tissue macrophages in the cochlea during postnatal development. *Hear Res* **365**, 110-126 (2018).
15. D. S. Sharlin, T. J. Visser, D. Forrest, Developmental and cell-specific expression of thyroid hormone transporters in the mouse cochlea. *Endocrinology* **152**, 5053-5064 (2011).
16. E. J. Son *et al.*, Developmental gene expression profiling along the tonotopic axis of the mouse cochlea. *PLoS One* **7**, e40735 (2012).
17. M. O. Trowe *et al.*, Loss of Sox9 in the periotic mesenchyme affects mesenchymal expansion and differentiation, and epithelial morphogenesis during cochlea development in the mouse. *Dev Biol* **342**, 51-62 (2010).
18. D. Phippard, L. Lu, D. Lee, J. C. Saunders, E. B. Crenshaw, 3rd, Targeted mutagenesis of the POU-domain gene Brn4/Pou3f4 causes developmental defects in the inner ear. *J Neurosci* **19**, 5980-5989 (1999).
19. J. C. Adams, Immunocytochemical traits of type IV fibrocytes and their possible relations to cochlear function and pathology. *J Assoc Res Otolaryngol* **10**, 369-382 (2009).
20. J. Chen, H. S. Shapiro, J. Sodek, Development expression of bone sialoprotein mRNA in rat mineralized connective tissues. *J Bone Miner Res* **7**, 987-997 (1992).
21. P. Moffatt *et al.*, Bril: a novel bone-specific modulator of mineralization. *J Bone Miner Res* **23**, 1497-1508 (2008).

22. V. E. Ramstad, A. Franzen, D. Heinegard, M. Wendel, F. P. Reinholt, Ultrastructural distribution of osteoadherin in rat bone shows a pattern similar to that of bone sialoprotein. *Calcif Tissue Int* **72**, 57-64 (2003).
23. N. G. Robertson *et al.*, Inner ear localization of mRNA and protein products of COCH, mutated in the sensorineural deafness and vestibular disorder, DFNA9. *Hum Mol Genet* **10**, 2493-2500 (2001).
24. W. Liu, A. Schrott-Fischer, R. Glueckert, H. Benav, H. Rask-Andersen, The Human "Cochlear Battery" - Claudin-11 Barrier and Ion Transport Proteins in the Lateral Wall of the Cochlea. *Front Mol Neurosci* **10**, 239 (2017).
25. M. Kwiatkowska *et al.*, The expression pattern and inhibitory influence of Tenascin-C on the growth of spiral ganglion neurons suggest a regulatory role as boundary formation molecule in the postnatal mouse inner ear. *Neuroscience* **319**, 46-58 (2016).
26. M. Downes, P. Koopman, SOX18 and the transcriptional regulation of blood vessel development. *Trends Cardiovasc Med* **11**, 318-324 (2001).
27. T. M. Williams, M. P. Lisanti, The caveolin proteins. *Genome Biol* **5**, 214 (2004).
28. T. Ito, N. Kurata, Y. Fukunaga, Tissue-Resident Macrophages in the Stria Vascularis. *Front Neurol* **13**, 818395 (2022).
29. X. Shi, Resident macrophages in the cochlear blood-labyrinth barrier and their renewal via migration of bone-marrow-derived cells. *Cell Tissue Res* **342**, 21-30 (2010).
30. Y. Igarashi, M. Y. Lee, S. Matsuzaki, Acid phosphatases as markers of bone metabolism. *J Chromatogr B Analyt Technol Biomed Life Sci* **781**, 345-358 (2002).
31. F. H. Drake *et al.*, Cathepsin K, but not cathepsins B, L, or S, is abundantly expressed in human osteoclasts. *J Biol Chem* **271**, 12511-12516 (1996).
32. W. Berger *et al.*, An animal model for Norrie disease (ND): gene targeting of the mouse ND gene. *Hum Mol Genet* **5**, 51-59 (1996).
33. U. F. Luhmann *et al.*, Fetal loss in homozygous mutant Norrie disease mice: a new role of Norrin in reproduction. *Genesis* **42**, 253-262 (2005).
34. I. Sorensen, R. H. Adams, A. Gossler, DLL1-mediated Notch activation regulates endothelial identity in mouse fetal arteries. *Blood* **113**, 5680-5688 (2009).
35. L. Madisen *et al.*, A robust and high-throughput Cre reporting and characterization system for the whole mouse brain. *Nat Neurosci* **13**, 133-140 (2010).
36. N. Harada *et al.*, Intestinal polyposis in mice with a dominant stable mutation of the beta-catenin gene. *EMBO J* **18**, 5931-5942 (1999).
37. K. Vikhe Patil, B. Canlon, C. R. Cederroth, High quality RNA extraction of the mammalian cochlea for qRT-PCR and transcriptome analyses. *Hearing Research* **325**, 42-48 (2015).
38. D. Bryant *et al.*, The timing of auditory sensory deficits in Norrie disease has implications for therapeutic intervention. *JCI Insight* **7** (2022).
39. M. D. Young, S. Behjati, SoupX removes ambient RNA contamination from droplet-based single-cell RNA sequencing data. *Gigascience* **9** (2020).
